# Molecular communications in complex systems of dynamic supramolecular polymers

**DOI:** 10.1038/s41467-022-29804-5

**Published:** 2022-04-20

**Authors:** Martina Crippa, Claudio Perego, Anna L. de Marco, Giovanni M. Pavan

**Affiliations:** 1 Department of Applied Science and Technology, Politecnico di Torino, 10129 Torino, Italy; 2 Department of Innovative Technologies, University of Applied Sciences and Arts of Southern Switzerland, Polo Universitario Lugano, 6962 Lugano-Viganello, Switzerland; 3 Department of Physics, Università degli Studi di Genova, 16100 Genova, Italy

**Keywords:** Self-assembly, Coarse-grained models, Supramolecular polymers, Statistical physics, Molecular dynamics

## Abstract

Supramolecular polymers are composed of monomers that self-assemble non-covalently, generating distributions of monodimensional fibres in continuous communication with each other and with the surrounding solution. Fibres, exchanging molecular species, and external environment constitute a sole complex system, which intrinsic dynamics is hard to elucidate. Here we report coarse-grained molecular simulations that allow studying supramolecular polymers at the thermodynamic equilibrium, explicitly showing the complex nature of these systems, which are composed of exquisitely dynamic molecular entities. Detailed studies of molecular exchange provide insights into key factors controlling how assemblies communicate with each other, defining the equilibrium dynamics of the system. Using minimalistic and finer chemically relevant molecular models, we observe that a rich concerted complexity is intrinsic in such self-assembling systems. This offers a new dynamic and probabilistic (rather than structural) picture of supramolecular polymer systems, where the travelling molecular species continuously shape the assemblies that statistically emerge at the equilibrium.

## Abstract (web summary)

The dynamic structure of supramolecular polymers is challenging to determine both in experiments and in simulations. Here the authors use coarse-grained molecular models to provide a comprehensive analysis of the molecular communication in these complex molecular systems.

## Introduction

Synthetic supramolecular polymers, composed of monomers that self-assemble in solution via reversible, non-covalent interactions, are important platforms for the development of advanced materials for many applications^1–4^. Such polymers are formed by monomeric units interacting via hydrogen bonding, solvophobic repulsion, *π*–*π* stacking, etc^1,5^. Thanks to such reversible interactions, supramolecular polymers bear unique dynamical properties at the equilibrium, which are at the basis of stimuli-responsive and adaptive features reminiscent of living materials^6–10^.

While, in principle, changing the monomer structure allows tuning the dynamic properties of supramolecular polymers, the complexity of these systems makes it often impossible to obtain clear monomer-assembly relationships. Various experimental approaches were used to characterise the structure of supramolecular polymers in solution^5,11–15^, most often providing average information on the entire solution, with limited detail on the distribution of aggregate sizes. Considerable efforts were spent also in characterising the dynamics of supramolecular polymers using, e.g., Förster Resonance Energy Transfer (FRET)^16,17^, Stochastic Optical Reconstruction Microscopy (STORM)^17–19^, or Hydrogen/deuterium exchange (HDX) mass spectrometry^20,21^. Typically, such approaches allow studying supramolecular polymer dynamics at the level of statistical ensembles or of individual assemblies (e.g., using bulky labelling groups, STORM detects supramolecular exchange with a resolution of ~20–50 nm)^17–19,22,23^. However, obtaining a clear understanding of the molecular processes that, in concert, define the dynamic nature of supramolecular polymers would ideally require a complete characterisation of such systems with submolecular resolution, which is mostly inaccessible via experiments.

Computational modelling recently emerged as a crucial tool to investigate the dynamic nature of supramolecular polymers. Stochastic polymerisation models^24^ have been employed to study the self-assembly of amyloid fibres^25^ or synthetic self-assembling systems, such as e.g., 1,3,5-benzenetricarboxamides (BTAs)^26^ or phenylenevinylenes^27^. Such models can predict the evolution of the aggregate population, resulting in polymerisation curves that are validated by fitting the signals detected in experiments^28–30^. At atomistic resolution, first-principles molecular dynamics (MD) simulations were recently demonstrated to be useful for the study of supramolecular polymers^17,31–35^. Coarse-grained MD (CG-MD) simulations have been also employed to study complex supramolecular polymer systems, reaching relevant space and time-scales to capture their dynamics^36–44^. In particular, combined with enhanced sampling methods, such models allow to study the molecular exchange between the supramolecular structures and their surroundings^22,32,45–48^, the exchange pathways^49^, and the response of these materials to external stimuli^50,51^. Such advanced computational approaches are mostly employed to study isolated assemblies in solution, whereas the dynamics of a system involving multiple assemblies (e.g., monomers/oligomers exchange between fibres, or fibres fragmentation and recombination) remains typically overlooked. To understand how supramolecular polymers communicate with each other in a realistic system, it is necessary to simulate multiple assemblies in dynamic, supramolecular equilibrium conditions, studying their complex, concerted behaviour with sufficiently high resolution.

Inspired by simulation studies in the field of colloids^52–54^, we here designed an inverse multiscale modelling approach, allowing us to study supramolecular polymers as complex molecular systems. Minimalistic CG models allow to reconstruct the network of molecular exchange events between the assemblies that populate these systems at the thermodynamic equilibrium. We can observe how these complex systems are composed of dynamic entities in continuous communication and exchange with each other and with the surrounding. Higher-resolution molecular models show how similar complexity is present also in realistic supramolecular polymer systems. This multiscale approach reveals the dynamic nature of supramolecular polymers, providing an exquisitely statistical view of these complex molecular systems.

## Results

### Dynamic equilibrium in a supramolecular system

**Fig. 1:**
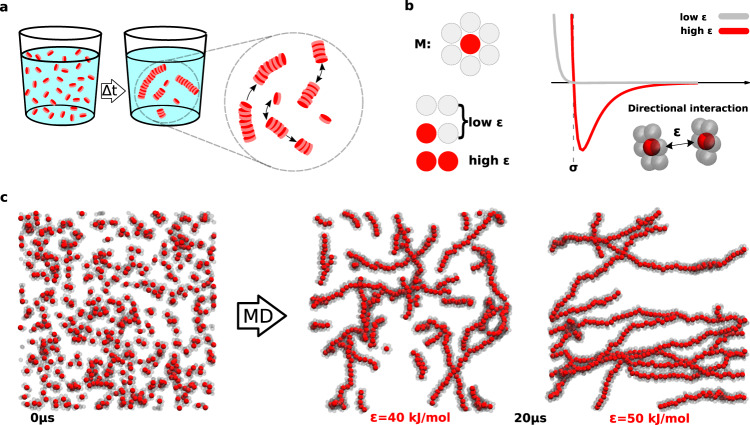
Minimalistic model of self-assembling monomers **M**. **a** Scheme for the self-assembly of monomeric **M** units into supramolecular polymers. At the thermodynamic equilibrium, the fibres exhibit a more/less pronounced dynamic behaviour, exchanging units and fragments with each other and with the external environment. **b** Structure and interaction of the minimalistic model: the **M** monomers interact directionally via attractive interaction between the central red beads. Weakly interacting beads (in grey) are added to screen the red beads and prevent lateral binding of the monomers (imparting directionality to the **M**–**M** interaction). The interactions in the model are defined by Lennard–Jones potentials. **c** CG-MD simulation snapshots of a model system composed of 500 initially randomly distributed monomers: the two snapshots at *t*_*CG*_ = 20 *μ*s refer to cases with the interaction strength between central beads is set at *ϵ* = 40 kJ mol^−1^ (centre) or *ϵ* = 50 kJ mol^−1^ (right), respectively, (both model systems start from the same initial condition, on the left).

In systems that self-assemble in solution, the formed assemblies reach a dynamic supramolecular equilibrium, characterised by continuous exchange of molecular units among assemblies and with their environment (Fig. 1a). To explore the factors that control such complex dynamics, we here employ a minimalistic model of a supramolecular polymer system, constituted by monomeric units that mutually interact in a directional, short-ranged and reversible way, spontaneously polymerising into one-dimensional fibres during MD simulations (Fig. 1).

The monomers (**M**) are composed of seven beads (Fig. 1b) disposed in a planar hexagonal shape (see Methods and Supporting Information, SI, for details), and interact mostly via the central beads, which are strongly attracted to each other. The six lateral beads are instead weakly interacting and, shielding the attraction between core beads at the monomer sides, impart directionality to the self-assembly. The non-bond interactions between beads are determined by Lennard–Jones (LJ) potentials, with fixed radius (*σ*) for all the beads, and two different values of interaction strength (*ϵ*): large (several *k*_*B*_*T*s) for the interaction between central beads, and small (a fraction of *k*_*B*_*T*), when lateral beads are involved (Fig. 1b). The dynamics of the system is modelled via Langevin equations of motion. The effect of the solvent is represented implicitly, i.e. the non-bond potential between the beads accounts for both solute-solute and solute-solvent interactions, while friction and fluctuations are rendered by Langevin dynamics. As the interaction between monomers is mainly governed by directional, specific attraction, this model mimics supramolecular polymers where (i) monomers self-assemble in a good solvent, and (ii) the monomer shape and features favour mutual directional aggregation^49^.

**Fig. 2:**
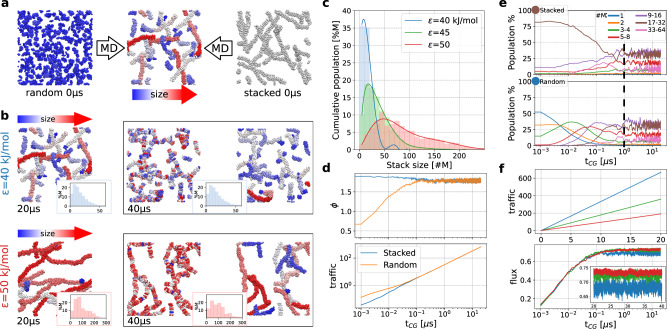
Dynamic equilibrium in a minimalistic self-assembling model. **a** CG-MD simulations of a model system composed of 500 **M** monomers, starting from randomly dispersed monomers (R case, left) or monomers pre-stacked into 20 equal fibres (S case, right). During CG-MD both systems relax to the same dynamical equilibrium state (centre: example snapshot). The assemblies are coloured based on their size (see colour bar). **b** CG-MD snapshots for the *ϵ* = 40 kJ mol^−1^ (top) and *ϵ* = 50 kJ mol^−1^ (bottom) systems. Left: snapshots at *t*_*CG*_ = 20 *μ*s (assemblies coloured according to their size). Centre: snapshots at *t*_*CG*_ = 40 *μ*s, where the monomer colouring used at *t*_*CG*_ = 20 *μ*s (left) is preserved - the colour reshuffling indicates molecular exchange after equilibrium is reached. Right: same snapshots as centre but assemblies recolored according to their current size (at *t*_*CG*_ = 40 *μ*s). The relative assembly-size distributions are reported in the insets. **c** Average distribution of aggregate sizes for different *ϵ* values at the equilibrium. **d** Average coordination number *ϕ* between the **M** centres (top) and cumulative molecular traffic^55^ (bottom) along CG-MD in the S and R cases. **e** Evolution of the assembly-size populations for the S (top) and R (bottom) cases. The brown and blue circles at *t*_*CG*_ = 0 indicate the initial population in the two cases. After *t*_*CG*_ = 1 *μ*s (vertical dashed black line) the two systems reach a comparable microscopic equilibrium state, and the populations plateau. **f** Molecular traffic and flux^55^ vs. time at different *ϵ* values.

We simulated 500 **M** monomers in a fixed cubic volume, at fixed temperature (*T* = 300 K). Starting from randomly distributed monomers, one-dimensional fibres spontaneously formed during CG-MD (Fig. 1c), reaching an equilibrium that depends on the stacking attraction between monomer cores (the interaction strength *ϵ* between the central beads). We first compared CG-MD simulations of the same system (*ϵ* = 40 kJ mol^−1^), starting from two different configurations (Fig. 2a), where the 500 **M** monomers are either randomly dispersed/disassembled (R) or pre-stacked into 20 equally-sized fibres (S). After a transient phase of the order of *μ*s both simulations converge to the same equilibrium state.

Figure 2b shows that, at the equilibrium, molecules are continuously exchanged between assemblies, even after the size-distribution has converged (insets of Fig. 2b). The average coordination between monomer centres (*ϕ*), indicates the stacking order of the system (*ϕ* = 2 signals perfectly stacked infinite fibres). Independently of the starting condition, the system converges to the same average structural/stacking order (Fig. 2d, top).

The dynamic nature of the supramolecular equilibrium state, qualitatively shown in Fig. 2b, is quantitatively analysed in Fig. 2c–f. We e.g., report the molecular traffic and flux^55^ along CG-MD (Fig. 2d(bottom), 2f(top), Supplementary Figs. 3–7): the traffic indicates the sum of molecular binding and unbinding events (per monomer) during the CG-MD, while the flux indicates the cumulative difference between binding and unbinding events (see Methods for details). After the equilibration stage (~1 *μ*s of CG-MD), the flux reaches a plateau while the traffic keeps increasing (see Fig. 2f). This means that, at the equilibrium, the average size of aggregates remains stable (binding and unbinding events balance), while monomers/oligomers continuously exchange among different constructs. This manifests the intrinsically dynamic character of these systems at the equilibrium. Both S and R cases converge to the same traffic and flux profiles, indicating that they have reached the same dynamical equilibrium state.

While these are averaged results, which pertain to the entire solution, the same holds also at a *microscopic* level, relatively to the size of individual assemblies that populate the system (Fig. 2e). After ~1 *μ*s of CG-MD, the assembly populations converge to nearly identical size distributions in both S and R cases, as these systems lose memory of the initial conditions and converge to the same dynamical equilibrium state. The dynamics of this model should be compared carefully with experiments. The minimalistic molecular representation and large monomer concentration of the simulations (~77 mM *vs*. typical experimental concentrations in the *μ*M scale) may impact the self-assembly pathway^39^, the size-distribution at the equilibrium, etc. Nonetheless, this minimalistic model demonstrates how changing key parameters affects the supramolecular dynamics, providing useful qualitative trends.

To investigate how the monomer–monomer interaction strength affects such collective structural/dynamical equilibrium, we compared simulations with increasing interaction strength (*ϵ*) between the monomer core beads: *ϵ* = 40 kJ mol^−1^, *ϵ* = 45 kJ mol^−1^ and *ϵ* = 50 kJ mol^−1^. As shown in Fig. 2b, at the dynamical equilibrium state the assembly size-distribution is globally conserved (insets), while molecular entities are continuously exchanged between the assemblies (details on the equilibration are reported in Supplementary Figs. 2–10).

The data show how changing the *ϵ* can entail a different equilibrium structure and dynamics (Fig. 2b, c, f). The size distributions of Fig. 2c indicate that longer fibres are formed when the directional interaction between the monomers is stronger. At the same time, the evolution of traffic shows that the the systems are increasingly dynamic when *ϵ* is decreased, even after the equilibrium is reached, as signalled by the convergence of the molecular flux (Fig. 2f).

Thus, our data support a general trend: increasing the monomer-monomer interactions produces longer fibres, but reduces the molecular exchange at the equilibrium (Fig. 2c, f, and Supplementary Figs. 2–12). Such behaviour fits qualitatively well with recent observations on, e.g., BTA systems, where increasing the number of carbon atoms in the monomer side-chains (favoring their self-assembly in aqueous environment) produces more persistent and less dynamical fibres in water^17,20,45,56,57^.

### Molecular communications in supramolecular polymer systems

The equilibrium dynamics of supramolecular systems involves a continuous exchange of molecular entities. Relevant questions concerning such communication between assemblies are: do all fibres exchange the same kind of fragments, or do they communicate in different ways (e.g., exchanging different species) depending on their size? In general, what factors control this inter-assembly communication?

**Fig. 3:**
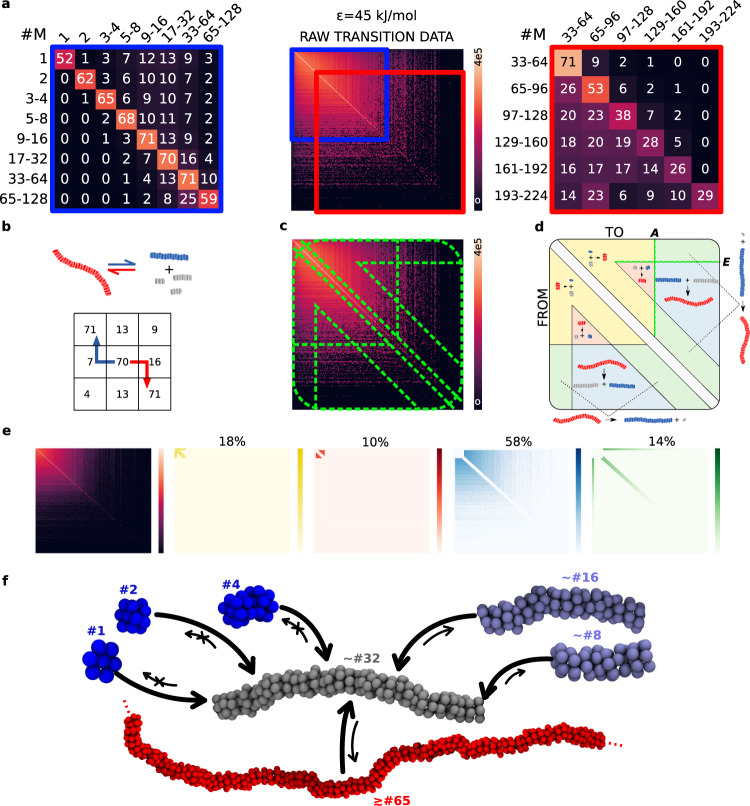
The dynamic nature of a supramolecular polymer system. Results are reported for the *ϵ* = 45 kJ mol^−1^ system (other cases are reported in the SI). **a** The transition matrix obtained from CG-MD with sampling interval Δ*τ* = 300 ps (centre). The left and right panels report two sub-regions of the transition probability matrix (red and blue rectangles). Here, the size of the aggregates are grouped for clarity. The numbers in the cells indicate the percentage probability (the 0s identify transitions with probability ≤0.5%, see Supplementary Fig. 13). The colouring of the matrix cells mirrors the entry values (with logarithmic scale for the raw transition data matrix). **b** Illustrative scheme interpreting the transition matrices in terms of polymerisation (red arrow) and depolymerisation (blue arrow) events. **c** Matrix partitioned in areas indicating the different polymerisation/depolymerisation mechanisms. **d** Diagram associating the different polymerisation/depolymerisation mechanisms to the different regions of the transition matrix. **e** Matrix counting the assembly transitions (left) decomposed into areas (as in **d**) identifying different classes of polymerisation/depolymerisation mechanisms (see Methods for details). **f** Dynamic interconnections between a 32 monomer aggregate (grey) and smaller (blue) or larger assemblies (red).

To study in detail what species are exchanged among the fibres at the equilibrium, we monitored the size of the assemblies that populate the systems over time, and tracked the transitions of each monomer among the different-size assemblies every Δ*τ* time interval. We analysed in this way the CG-MD trajectories at equilibrium (*t*_*CG*_ = 20 − 40 *μ*s) with a sampling time interval of Δ*τ* = 300 ps, frequent enough to reduce the probability of assemblies undergoing multiple binding/unbinding events during Δ*τ* (see Methods for details). We thus obtained transition matrices (Fig. 3a, centre) counting how many times a monomer is exchanged from an assembly of size *i* (matrix row index) to another of size *j* (column index). Normalising the rows of these matrices to 1, we obtain the transition probability matrices of, e.g., Fig. 3a (left and right). Here, the numbers on the diagonal indicate the probability that a monomer inside an assembly of size *i* remains in that assembly during a Δ*τ* interval. The off-diagonal entries are indicative of the probability that a monomer in an assembly of size *i* is involved in a transition, becoming part of a larger (*i*-to-*j*, where *j* > *i*) or smaller (*j* < *i*) assembly during Δ*τ*. It is worth noting that all raw matrices are symmetric (see Supplementary Figs. 21–23), i.e., binding and unbinding events are balanced (as expected for dynamic systems at the equilibrium). The symmetry is instead not preserved in the transition probability matrices, as a consequence of the normalisation.

Figure 3 reports the transitions detected for the *ϵ* = 45 kJ mol^−1^ system (see SI for data on the other systems and additional details). Two regions of the transition probability matrix are reported in Fig. 3a (left and right panels), identifying respectively the dynamics of smaller or larger aggregates. As summarised in the scheme of Fig. 3b, the off-diagonal pathways on the right (red arrow) signal polymerisation events, while the left ones (blue arrow) signal depolymerisation events. Noteworthy, at the equilibrium, small assemblies are more likely to aggregate into larger assemblies, rather than to disassemble—the probabilities are larger on the right-side of the diagonal (Fig. 3a, left). This is the case up to assemblies of size ~33−64 monomers for the *ϵ* = 45 kJ mol^−1^ system (but that depends on the *ϵ* value). As the range increases, the fibres become inherently less stable, and fragmentation becomes more likely than growth (Fig. 3a: right and b). The tendency to self-assembly is thus contrasted by the increasing probability of fragmentation. These competing factors determine the dynamic equilibrium in the monomer exchange. When *ϵ* is decreased (40 kJ mol^−1^), the equilibrium shifts to smaller aggregate sizes (Supplementary Fig. 14a: highest probability for ~5−16 monomers). Vice versa, for stronger monomer-monomer interactions (*ϵ* = 50 kJ mol^−1^) the equilibrium moves towards longer fibres (Supplementary Fig. 14b: highest probability for ~65−128). We refer to logarithmic ranges of fibre sizes, as we aimed at obtaining a qualitative picture of how the equilibrium dynamics shapes the entities that are most probably observed, keeping a high focus on smaller species and lower detail for larger assemblies (for comparison, the same data are reported using a linear size-grouping in Supplementary Fig. 31).

Comparing the transition matrices of the three systems, we also observe that, as *ϵ* increases, it becomes less likely to observe monomers and small aggregates. At *ϵ* = 40 kJ mol^−1^, the disassembled monomers have a ~60% probability to remain isolated at every Δ*τ* (Supplementary Fig. 14a: left). The probability drops to ~52% and ~49% for the systems with *ϵ* = 45 and *ϵ* = 50 kJ mol^−1^ cases (Fig. 3a: left and Supplementary Fig. 14b: left, respectively).

Overall, these transition matrices (Fig. 3a and Supplementary Fig. 14) provide also insights into the prevalent mechanisms by which the assemblies exchange molecular units/fragments, communicating with each other. Comparing between the different systems, we can assess how changes in the system parameters may transform such communications. The fibres can either elongate by acquiring monomers (or oligomers), or by coalescence with similar-size assemblies. Similarly, fibre depolymerisation may occur via monomer (or oligomer) exchange with the solution, or via fragmentation into shorter fibres. The scheme of Fig. 3d identifies those regions of the transition matrix (i.e., Fig. 3c) that refer to such different classes of events: involving the exchange of small size aggregates (yellow and green areas) or the coalescence/fragmentation of medium/large constructs (red and blue areas). For simplicity, the representation in Fig. 3d shows only “binary” events, neglecting those cases in which a fibre fragments into more than two sections, or multiple fragments combine into a single assembly. These events can be considered rare given the short sampling interval Δ*τ* (see SI for details). We quantified the statistical relevance for the different mechanisms represented in Fig. 3d. To this end, we first chose two characteristic aggregate sizes that delimit the matrix areas in the scheme: the first, *A*, discerns between *medium* and *large* assemblies, and is chosen as the average aggregate size at the equilibrium *A* = 〈size〉: (e.g., ~21 monomers in the *ϵ* = 45 kJ mol^−1^ system). The second parameter, *E* = 〈size〉/5 ~ 4, distinguishes between *medium* and *small* assemblies (Fig. 3d). Using these parameters, we observe that, for the *ϵ* = 45 kJ mol^−1^ system, ~58% of the total transition events is imputable to the coalescence/fragmentation of the large fibres (*i.e*., it involves transitions where the size of both the starting and final fibres is >*A* - see Fig. 3d, e: blue area). In a similar way, we calculated that ~18% of the events consist in the elongation/shortening of smaller fibres (of size < *A*) via binding/unbinding of monomers (or small oligomers of size < *E*: yellow area of Fig. 3e). Elongation/shortening of long fibres (size > *A*) via binding/unbinding of monomers/small oligomers impacts for ~14% of all the events (Fig. 3e: green area), while 10% of transitions involves the coalescence/fragmentation of medium-size fibres (*E* < size < *A*, red area). While the blue areas contribute the most to the dynamics of this system, also events involving small/medium species appear with relevant probabilities. While the weight of different mechanisms may change depending on the choice of *A* and *E*, this analysis provided rather robust evidence that all four mechanisms shown in Fig. 3d contribute substantially to the system dynamics (see SI for additional details).

We also observed that the exchange of monomers/oligomers between the fibres and the environment occurs mostly via the fibre tips. Exchange of monomers/oligomers from the fibre-bulk was detected with an occurrence of <0.001% of the binding/unbinding events (see Supplementary Figs. 21–23 for details). Generating well-ordered fibres via directional interactions (negligible solvophobic interactions), the **M** model mimics the case of supramolecular polymers in a good solvent, where molecular exchange occurs preferentially from the fibre tips. Monomer exchange from the fibre bulk is instead typical of systems dominated by non-directional/solvophobic effects, where defects along the fibres govern the monomer dynamics^45,49^.

From the transition probabilities, it is possible to build diagrams of the dynamic interconnections between assemblies in these systems (see e.g., Fig. 3f). The results obtained with such a minimalistic model highlight the complexity of the dynamic equilibrium in elementary supramolecular systems. It is interesting to investigate if such complexity is encountered also in more realistic supramolecular polymer models, and what aspects are unavoidably oversimplified and overlooked.

### Analogies with realistic supramolecular polymer models

**Fig. 4:**
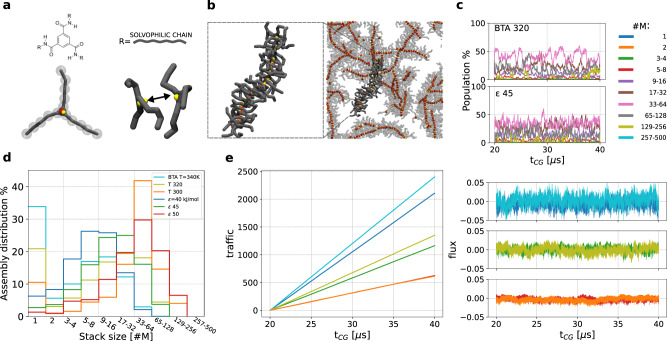
Coarse-grained model of self-assembling supramolecular polymers. **a** Molecular structure of a **BTA** monomer core (top), which can be decorated with generic (e.g., solvophilic) side chains. Bottom: CG model of a **BTA**-like solvophilic monomer (left), which interacts directionally with monomers of the same specie (right). **b** Snapshot of **BTA** fibres formed spontaneously after *t*_CG_ = 20 *μ*s of CG-MD, starting from 500 dispersed **BTA** monomers. A single fibre is highlighted and reported in the frame on the left. **c** Time evolution of the assembly-size populations (in percentage of monomers) for the **BTA** model at *T* = 320 K (top) and **M** model with *ϵ* = 45 kJ mol^−1^ (bottom) at the equilibrium. **d** Distribution of assemblies of different sizes (percentage over the average number of assemblies) for different **BTA** and **M** systems. **e** Cumulative molecular traffic (left) and flux (right)^55^ vs. time for different **BTA** and **M** systems at the equilibrium (same colours of (**d**)).

To increase the chemical relevance of our study, we then compared the behaviour of the **M** model with a higher-resolution supramolecular polymer model. Building on our simulations on BTA supramolecular polymers^38,39,45,58^, we recently developed a CG model that, while being consistent with BTA behaviour, is representative of a larger class of supramolecular polymers, formed by monomers with three symmetric side-chains connected to a stacking centre^49^. In this CG model, the stacking centre is composed of a central bead and a charged dipole. The dipoles of different monomers interact with each other, reproducing a directional interaction consistent with that of BTA motifs. Three flexible side-chains, each composed of five CG beads (Fig. 4a, bottom) are symmetrically connected to the monomer centre. The side-chains beads are solvophilic, so that this model is consistent with BTA supramolecular polymers (and analogous supramolecular motifs) in a good solvent (similarly to the **M** minimalistic model described above)^49^. Initially developed as explicit solvent model^49^, we here re-parametrised this CG model to behave consistently in implicit solvent (see Methods and SI for details). This allowed us to simulate a large number of monomers for relatively long timescales, obtaining results comparable to those obtained with the **M** model. In the following we will refer to this model as “**BTA**”.

The **BTA** monomers are intrinsically different from the **M** units. In fact, while the flat symmetry and directional interaction between the **BTA** cores produce an ordered monomer stacking as in the **M** model, the higher-resolution description of the **BTA** monomers accounts (to some extent) for the effect of molecular flexibility (whereas the **M** units are rigid). Investigating how similar is the supramolecular equilibrium of the **BTA** model to that of the **M** model could provide insights on how molecular flexibility impacts the dynamics of the assembled system. Also, it could reveal to what extent the complexity of a realistic system is lost when considering a minimalistic representation such as that of **M** model.

Also in this case, we simulated the self-assembly of 500 initially dispersed **BTA** monomers in a cubic simulation box, such that the monomer concentration was identical to that of the **M** simulations (see Methods). In this case, we conducted CG-MD simulations at three different temperatures: *T* = 300 K, *T* = 320 K and *T* = 340 K, as increasing/decreasing the temperature has the effect of weakening/strengthening the self-assembly propensity. Similarly to the **M** model, the **BTA** monomers reached the equilibrium forming well-ordered fibres of diverse sizes during CG-MD (Fig. 4b, right). The CG-MD trajectories of these simulations were then analyzed as done for the **M** model, comparing the structural and dynamical behaviour of the different models at the equilibrium. Figure 4c shows that, at the equilibrium, similarly-sized aggregates populate the system in the **BTA**-like system at *T* = 320 K and the **M** system with *ϵ* = 45 kJ mol^−1^ (see Supplementary Fig. 20 for the other systems). This similarity is confirmed by Fig. 4d, showing the average percentage of different-size assemblies that populate the systems in equilibrium conditions (see also Fig. 4d, Supplementary Figs. 18 and 19).

Comparing these simulations also demonstrates that changing the temperature in the **BTA** systems has similar effect on the aggregate-size distribution than varying *ϵ* in the **M** model. Increasing the **BTA** system temperature to 340 K generates an equilibrium size distribution more similar to that obtained in the **M** system at *ϵ* = 40 kJ mol^−1^ (Fig. 4d). In both cases, relatively small aggregates (average size < 10 monomers) are observed, with significant free-monomer populations, up to ~34% **BTA** case and ~6% for the **M** model (more comments on this aspects in the next section). Although these model systems are considerably over-concentrated compared to realistic dilute conditions, thus favoring monomer aggregation, these data suggest a fairly limited tendency to self-assembly. These results are in qualitative agreement with experimental evidence showing a critical transition temperature for, e.g., the **BTA** motif at ~70 ^∘^C (~343 K), above which **BTA** self-assembly is hindered^38,49,57^. Analogous qualitative similarities hold also when the **BTA** model at *T* = 300 K and **M** model with *ϵ* = 50 kJ mol^−1^ are compared, as the spontaneous formation of longer fibres is favoured in both cases (Fig. 4d).

The molecular traffic and flux data of Fig. 4e show that, globally, the temperature variation affects also the equilibrium dynamics of the **BTA** systems similarly to what the variation of *ϵ* does in the **M** system. In general, the collective behaviour of these two models exhibits significant analogies on an ensemble (average) level. However, deeper-level questions arise when comparing the **M** and **BTA** systems at a microscopic level: how similar is the molecular exchange and communication dynamics among the self-assembled fibres in these two models of complex systems?

### Mechanisms of communication between supramolecular polymers

**Fig. 5:**
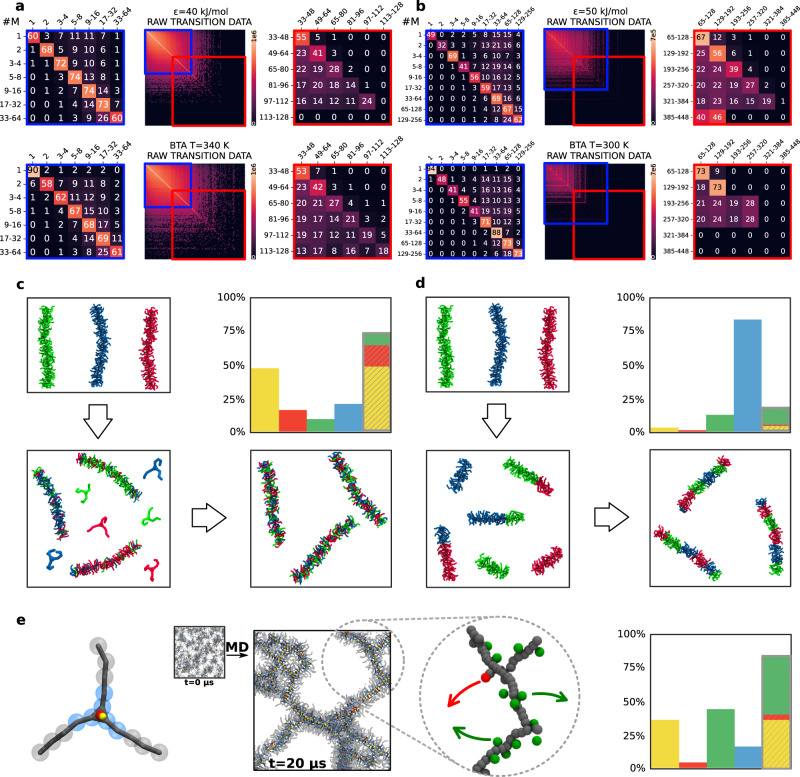
Equilibrium dynamics in supramolecular polymer systems. **a** Raw transition matrix (centre) and transition probability sub-matrices (left and right, as in Fig. 2a), comparing the **M** system with *ϵ* = 40 kJ mol^−1^ (top) and the **BTA** system at *T* = 340 K (bottom). **b** Same as (**a**), comparing the **M** system with *ϵ* = 50 kJ mol^−1^ (top) and the **BTA** system at *T* = 300 K (bottom). **c**, **d** Illustrative schemes of the mechanisms of inter-assembly communication. When the interaction between the self-assembling units is weaker (lower *ϵ*, or higher *T*), the fibres preferably communicate with each other exchanging monomers or relatively small fragments. When the interaction between self-assembling units is stronger (higher *ϵ*, or lower *T*), the inter-assembly communication proceeds mostly via large fibre fragmentation and coalescence. The histograms indicate the incidence of the four communication mechanisms detailed in the text. The same colour coding of Fig. 3e is used. **e** Comparison with a model of water-soluble BTA supramolecular polymers (**BTA**_*w*_: where the monomers are amphiphilic, and solvophobic effects are non-negligible). Left: structure of the **BTA**_*w*_ monomer: the monomer cores (blue beads) attract each other with interaction strength *ϵ* = 4 kJ mol^−1^, to reproduce the solvophobic effect. Centre-left: snapshot of **BTA**_*w*_ fibres formed spontaneously after *t*_CG_ = 20 *μ*s of CG-MD (starting from 500 dispersed **BTA**_*w*_ monomers, small inset). Centre-right: detail of the **BTA**_*w*_ fibres, highlighting the presence of defects all along the fibres backbone (the spheres represent the centres of the monomers, bulk defected domains are highlighted in green, defected domains akin to fibre tips are highlighted in red—see also Supplementary Fig. 32). Mechanism histogram, showing that the fibres preferably communicate with each other exchanging monomers or relatively small fragments.

From the CG-MD simulations, we computed the transition matrices for the **BTA** systems in the same way as for the **M** ones. From Fig. 5a, b, it appears clear that analogies are observed also in what pertains the molecular exchange between the assemblies. Comparing the **M** system with *ϵ* = 40 kJ mol^−1^ and the **BTA** system at *T* = 340 K, we observe that the transition matrices look qualitatively similar. Peculiar, yet relevant differences can be nonetheless noticed. In particular, the free disassembled monomers at the equilibrium are more persistent in time in the **BTA** system with respect to the **M** system. Observing free monomers in solution is relatively more likely in the **BTA** than in the **M** systems (Figs. 4d and 5a, b). Noteworthy, this does not mean that the self-assembly is less favoured in the **BTA** system than in the **M** model - the probability associated to larger assemblies is rather similar in the two systems (e.g., ~73% for 17−32 monomers in the **M** system with *ϵ* = 40 kJ mol^−1^*vs*. ~  69 % in the **BTA** system at *T* = 340 K). In conditions where self-assembly is more favoured (e.g. **M** with *ϵ* = 50 kJ mol^−1^ and **BTA** at *T* = 300 K), large fibre sizes are even more persistent in time in the **BTA** than in the **M** system, while the same holds also for the monomers. This suggests that the free-energy landscape for monomers self-assembly differs between the **BTA** and the **M** system. In particular, consistent with the experimental evidence, the **BTA** systems show the characteristic behaviour of cooperative self-assembly, where a critical-size nucleus must be overcome to self-assembly, and where the presence of monomers along with longer fibres is expected at the equilibrium^26,32,38,59–61^. In this perspective, the assembly-size distribution in Fig. 4d and probability matrices in Fig. 5a, b depict **M** as a predominantly isodesmic system. The increased survival of disassembled monomers in the **BTA** model can be imputed to the flexible structure of the monomer. In such a model, the side-chains can wrap around the **BTA** centres, minimising the residual solvophobicity of the monomer (cores), and generating a globular and isotropic solvated state of the monomers. Such effect is absent in the **M** monomers due to their flat and rigid structure. The difference between the two models becomes less relevant when aggregates are considered (negligible for size >3−5 monomers), as in larger **BTA** assemblies the monomers can optimise their interactions collectively, forming ordered conformations in which the side-chains are extended and do not interfere with the core–core interactions^32,38^. Such evidence allows us to draw the following considerations. First, in such systems the cooperativity in the self-assembly seems to emerge from competing effects: the interaction between the monomers, favoring self-assembly, vs. the flexibility of the molecular structure, which determines differences between the configuration of monomers in the disassembled and in the assembled states, due to molecular movements and to the presence of residual (even minimal) solvophobic effects. Second, in the case of rather complex and flexible monomers, a minimalistic description of their structure may determine an over-approximated representation of self-assembly, impairing a correct modelling of cooperativity in self-assembly.

Figure 5b reports the transition matrices of the two model systems with the strongest self-assembling drive, namely the **M**s at *ϵ* = 50 kJ mol^−1^ and the **BTA**s at *T* = 300 K. We note that the molecules/fragments are exchanged between the various size assemblies in a similar way in both systems. In these conditions, the monomers (when they do not remain disassembled) tend to flow toward the most stable aggregates in the systems (33−64 and 65−128-mers). At larger sizes, this is again contrasted by the fibre tendency to fragmentation. Analogous similarities hold also for the cases of **M** with *ϵ* = 45 or *ϵ* = 40 kJ mol^−1^ and **BTA** at *T* = 320 or *T* = 340 K, respectively (see Fig. 5a, and Supplementary Fig. 24).

We then quantified the statistical weight of the different exchange mechanisms in the global equilibrium dynamics of the systems (see Methods for details). We classified the events tracked in the matrices as done in Fig. 3e (to ease the comparison, we used the same threshold sizes *A* and *E* defined for the **M** model, also for the **BTA** systems, complete data in Supplementary Fig. 25). To simplify the analysis, we now group all the exchange events under two main inter-assembly communication mechanisms: (i) via exchange of small entities (size<*E*) and fragmentation/coalescence of medium-size aggregates (*E* < size < *A*), or (ii) via fragmentation or coalescence of large molecular entities (of size > *A*). Referring to the illustrative diagram of Fig. 3d, (i) encompasses all events in the yellow, red and green areas, while (ii) identifies the events pertaining to the blue area. According to such a classification, this analysis estimates that ~74% of the exchange events in the **BTA** system at high-temperature (*T* = 340 K) involves only monomers and oligomers, while inter-fibres communication via fragmentation/coalescence contributes by ~26% (see Fig. 5c: grey vs. blue columns in the inset). Decreasing the temperature to *T* = 320 K, we observe an intermediate behaviour. Under such conditions, the dynamics of the system is controlled by exchange of both small (~60%) and large species (~40%—see Supplementary Fig. 25). The exchange of monomers and smaller fragments becomes then less relevant in the inter-fibre communications at *T* = 300 K for the **BTA** system. In such conditions, our simulations indicate that small exchanging species give a minor contribution to the molecular communication in the system, while fragmentation and fusion of longer fibres become dominant (Fig. 5d: grey *vs*. blue columns at ~17% vs. ~83%, respectively). A similar scenario is observed also for the **M** system with *ϵ* = 50 kJ mol^−1^ (Supplementary Fig. 25). Additional tests changing the threshold parameters used for these analyses demonstrate the robustness of the obtained results and the general validity of such evidence (see Supplementary Fig. 27).

The **M** and **BTA** systems appear similar also for what pertains to the dominant mechanisms of communication between assemblies in the systems. While the statistical weights discussed above may depend, to some extent, on the molecular concentration, the obtained trends provide solid, general-level insights. Our results reveal that the dominant mechanisms by which the fibres communicate in such complex systems may change as the interaction strength between the monomers varies (either by changing the *ϵ* or the *T*). This is captured in a similar way by both the minimalistic, rigid **M** model and by the more chemically-relevant, flexible **BTA** model. In this sense, it is worth pointing out that the results discussed herein hold for supramolecular polymers composed of monomers that can be effectively modelled as globally solvophilic units, the self-assembly of which is dominated by directional interactions. Our data show that in such a case, these systems are populated by relatively straight and ordered fibres that communicate with each other mainly via their tips (via monomer/oligomer exchange or fragmentation/recombination - see Supplementary Figs. 21–23), consistently with recent modelling studies^49^. This is also consistent with the available experimental evidence on, e.g., BTA^60,62^, porphyrin-based^46^, or NDI-based^22^ supramolecular polymers in good solvents, which prove how such ideal self-assembling conditions can be exploited to control, e.g., the formation of self-sorted, alternated, or block supramolecular copolymers^22^, or even to control the length of the supramolecular polymers in the system^63^.

Instead, when the monomers are more solvophobic, and non-directional interactions become non-negligible, the dynamics of the system might change, with consequences on the exchange kinetics, pathways and mechanisms, as indicated by the available experimental and computational evidence on, e.g., water soluble BTA supramolecular polymers^16–18,20,32,45,49,50,58^. In order to compare our modelling results with the available experimental and computational evidence, we extended our analysis to an additional case study. We designed a variant of the **BTA** model, named **BTA**_*w*_, that reproduces the self-assembly of monomers with a solvophobic core and amphiphilic side-chains, and in particular of the water-soluble BTA monomers recently studied both experimentally and computationally^16–18,20,32,45,49,50,58^ (see the Methods section and Fig. 5e and Supplementary Fig. 32a for details). The **BTA**_*w*_ monomers have the same shape and structure of the **BTA** ones, but they self-assemble via a combination of directional and non-directional (solvophobic) interactions. As for the **BTA** case, the **BTA**_*w*_ model has been optimised to behave consistently with a previously validated explicit-solvent model (see Methods and Supplementary Fig. 32b–e), that can reliably capture the key features of water-soluble BTA supramolecular polymer systems^49^. Analysis of CG-MD simulations of 500 **BTA**_*w*_ monomers at *T* = 300 K shows formation of fibres rich of structural defects (see Fig. 5e and Supplementary Fig. 32c, d). This is consistent with computational evidence from high-resolution molecular models^45,58^. In particular, it has been demonstrated how such bulk defects work as exchange “hot-spots”, which trigger molecular exchange from all along the length of the fibres^49^, consistently with the available experimental evidence on water-soluble BTA fibres^18^. Analysis of molecular exchange among the assemblies that populate the **BTA**_*w*_ system indicates that the communication between the **BTA**_*w*_ fibres is mainly controlled by the exchanges of monomers and small molecular species (which contribute ~84% of the transition events—see Fig. 5e and Supplementary Fig. 32f, g). The fragmentation/recombination of large fibres contributes only by ~16% of transition events, being much less prominent than in the **BTA** case at *T* = 300 K (~80%). This is also consistent with, e.g., STORM experiments, which show no evidence of fragmentation/recombination in water-soluble BTA fibres^18^, while this becomes more prominent in BTA supramolecular polymer systems in organic solvents^28,62^.

The global and microscopic similarities and the analogous scaling behaviour exhibited by these different models (Fig. 5, Supplementary Figs. 27 and  32) prove how even relatively simple self-assembling systems possess a rather complex dynamic character at the equilibrium. The obtained results demonstrate however that, not only the average assemblies sizes, but also the dynamic communication between aggregates that are formed at the supramolecular equilibrium is regulated by environmental variables (such as, e.g., temperature) as much as by molecular features of the self-assembling building blocks (e.g., monomer structure, interaction strength, etc.). This underlines the importance to move the attention from the structure of the fibres and from the monomers as individual entities, to the evaluation of the complexity that emerges in the system considered as a whole.

## Discussion

Supramolecular polymer systems are characterised by a dynamical equilibrium which confers to these materials an innate dynamic character and interesting bioinspired properties. The behaviour of such complex systems is typically hard to understand, as their properties are controlled by molecular factors as much as by the collective dynamic behaviour of the self-assembling entities that populate them. Studying the intricate network of collective interactions between the entities present in the systems with sufficient resolution to uncover the key molecular factors that control this complex dynamic behaviour is a non-trivial but crucial challenge. Here we designed a molecular simulation approach which allows us to reach this goal. Employing coarse-grained models and molecular simulations of systems containing a large number of interacting monomers, we can study the structural and dynamical features of supramolecular polymer model examples at the thermodynamic equilibrium.

Our approach allows us to track the dynamic exchange events that occur in these systems. In this way, we can analyse in detail the molecular exchange processes that govern the dynamic equilibrium of supramolecular systems, unveiling details that cannot be captured with single-fibre simulations or stochastic models, studying supramolecular polymers on a purely average level. Our modelling approach provides insights on how the assemblies communicate with each other, what are the exchange mechanisms, and how it could be possible, in principle, to control the collective behaviour of these complex systems.

The results that we obtained change the usual way we look at supramolecular polymers from an average, macroscopic and structure-based, to a more dynamical, collective and microscopic point of view. The attention is moved to the dynamic behaviour of the entities that statistically populate the system, and to the manner they dynamically and collectively communicate in what *de facto* is a complex molecular system. In such an exquisitely dynamical perspective, the supramolecular fibres that spontaneously form in the system are not the main subject, but they emerge statistically as a consequence of the dynamic behaviour of monomeric entities that are continuously exchanged in-and-out the assemblies.

The presented modelling strategy holds considerable potential for building relationships between environmental and structural changes on how the assemblies dynamically communicate with each other in such complex molecular systems. Also, the good agreement between these minimalistic models and the available experimental and computational evidence suggests the opportunity for building inverse multiscale modelling approaches capable of providing precious information to program the molecular communications in such complex systems via rational design/customisation of the constitutive self-assembling units.

We believe that the approach presented herein can be applied to study a variety of supramolecular polymers as well as other types of dynamic self-assembling systems, and to explore routes to control the dynamical behaviour of such complex systems by acting on the collective communication between their constitutive building blocks.

## Methods

### Minimalistic M model

The interacting hexagonal unit **M** is composed of seven beads, six shielding beads at the vertices of a regular hexagon and a central core bead, as depicted in Fig. 1. The flat hexagonal geometry of the **M** monomer model is imposed by bonded interactions defined by harmonic bonds: the force constant between the nearest neighbour beads is 20,000 kJ mol^−1^ nm^−2^ and the equilibrium length is 0.47 nm. In order to keep the **M** hexagons planar, each shielding bead is also connected with the one at the opposite vertex of the hexagon by a harmonic bond with equilibrium length 0.94 nm and force constant of 15,000 kJ mol^−1^ nm^−2^. The non-bonded interactions between beads are defined by Lennard–Jones (LJ) potentials, with constant *σ* = 0.47 nm and variable interaction strength *ϵ*. All the shielding beads interact weakly with each other and with the core beads (*ϵ* = 0.2 kJ mol^−1^), while the interaction between core beads are stronger, with an interactions strength that varies across simulated systems (40 kJ mol^−1^, 45 kJ mol^−1^ or 50 kJ mol^−1^). Such LJ parameters, plus the geometry of the **M** monomers, determine the tendency of the **M** monomers to interact directionally. All the model settings and parameters used herein are provided in the SI (in .GRO and .ITP GROMACS format).

### BTA models

The minimalistic **BTA** model employed herein is the implicit-solvent version of the *Fibre* **1** molecular model developed recently in Ref. ^49^. The topology of the monomer is identical, while the bonded and non-bonded interaction parameters have been adapted to work consistently in the absence of explicit-solvent molecules. This model is representative of threefold symmetric monomers (having three solvophilic arms that surround a planar core) which interact directionally with each other in a good solvent (including, but not limited to, e.g., BTA monomers with alkyl side chains immersed in organic solvents, etc.). Initially built starting from the topology of fine-grained MARTINI-based CG BTA-C6 monomers^38^, the **BTA** model has a more abstract and general structure, optimised to obtain a behaviour consistent with that of higher-resolution models^49^. The **BTA** monomer is composed by 18 beads of three different types (see Fig. 4a). At the centre of the molecule there is a CG-bead (Fig. 4a: in red), containing a rigid central dipole composed of two small, charged beads of *q* = ± 1.4 *e* (in yellow), oriented along the monomer axis. Three arms originate from the central bead, which are composed of 5 CG-beads, linearly connected to form a three-armed monomer. The intra-molecule bonded interactions are modelled via harmonic bond and angle potentials, the parameters are reported in the SI. The core and arm CG beads interact via Lennard–Jones (LJ) potential: the interactions between the beads of the side-chains have *ϵ* = 1 kJ mol^−1^ and *σ* = 0.47 nm, the interactions between the side-chain and core beads have *ϵ* = 0.5 kJ mol^−1^ and *σ* = 0.47 nm, while *ϵ* = 2.5 kJ mol^−1^ and *σ* = 0.47 nm for the core-core interactions. The charged beads that determine the central dipole have no LJ interactions, and interact with each other electrostatically, imparting directionality to the monomer-monomer interaction. The **BTA**_*w*_ model, representative of self-assembling amphiphilic monomers, is parametrised to behave as the implicit solvent version of the *Fibre* **3** model, studied in Ref. ^49^, which was optimised to behave consistently with higher-resolution molecular models which can reproduce the behaviour of, e.g., water-soluble BTA supramolecular polymers^38,45,49^. In practice, it is structurally identical to the **BTA** model, with the exception of the LJ interaction strength *ϵ* between the 6 central beads (highlighted in blue in Fig. 5e and Supplementary Fig. 32a) of the monomer, which is increased from 1 to 4 kJ mol^−1^. The correspondence between the implicit solvent models **BTA** and **BTA**_*w*_ with the explicit solvent BTA-like models of Ref. ^49^ (namely *Fibre* **1** and **3**) is manifest by the structural characterisation shown in Supplementary Fig. 32e. Complete details on the models and force field parameters used for the **BTA** and **BTA**_*w*_ monomer models are provided in the SI (in .GRO and .ITP GROMACS format).

### CG-MD simulations

The CG-MD simulations of the **M**, **BTA** and **BTA**_*w*_ models were carried out using the GROMACS software^64^ (versions 2018.6 and 2020.2). All the MD simulations were performed in NVT conditions, using a constant volume for the simulation box of 22.056 × 22.056 × 22.056 nm^3^, with periodic boundary conditions, a constant number of molecules *N* = 500, and a constant temperature. This corresponds to a monomer density of 0.0466 nm^−3^, equivalent to a concentration of ~77 mM. The only exception are the 40 monomers CG-MD simulations of **BTA** and **BTA**_*w*_ models (Supplementary Fig. 32c–e), which were performed at a density of 0.0036 nm^−3^, to enable the comparison with the simulations of Ref. ^49^. The temperature was set to *T* = 300 K, in the **M** and **BTA**_*w*_ model, and at different values (see main text) for the **BTA** model. The systems were simulated in implicit-solvent via Langevin dynamics, accounting for the friction of the solvent and thermal fluctuations of the system. We used the stochastic dynamics (sd) integrator, setting the inverse of the friction constant to tau-t = 0.1 ps. tau-t also sets the coupling with the random force term, that determines the temperature of the system. The time step was set at Δ*t* = 20 fs for the **M** model and at Δ*t* = 15 fs for the **BTA** and **BTA**_*w*_ model simulations. The non-bonded interaction potentials were truncated and shifted at *r*_*c*_ = 1.1 nm.

The equilibration phase of the **M** model was studied over six sets of simulations, *ϵ* = 40 kJ mol^−1^ (Fig. 2d, e, Supplementary Figs. 2, 3 and 8)*ϵ* = 45 kJ mol^−1^ (Supplementary Figs. 4, 5, and 9) and *ϵ* = 50 kJ mol^−1^ (Supplementary Figs. 6,  7 and 10) starting from either random (R) or stacked (S) configurations—*i.e.*, starting from *N* = 500 randomly distributed monomers (R), or arranged in 20 pre-stacked fibres composed of 25 monomers each (S). Each of these simulations are 20 *μ*s long and sampled every ns.

The analysis at the equilibrium of the **M** model has been performed for three different simulations, with *ϵ* = 40 kJ mol^−1^, *ϵ* = 45 kJ mol^−1^ and *ϵ* = 50 kJ mol^−1^ (Figs. 2c, f, 3, Supplementary Figs. 12 and 15). For each system we performed a total of 40 *μ*s of CG-MD, starting from randomly dispersed monomers. The first 20 *μ*s of CG-MD are considered as an equilibration stage (corresponding to the R simulations mentioned before), while the trajectory 20 to 40 *μ*s is employed for the equilibrium studies. During this second part of the CG-MD we sampled the conformations every 300 ps.

For the **BTA** model, we performed three sets of simulations at different temperatures: *T* = 340 K, *T* = 320 K and *T* = 300 K (Figs. 4, 5, Supplementary Figs. 17,  24,  25, and 27). As in the **M** model, for each system we performed a total of 40 *μ*s of CG-MD, starting from randomly dispersed monomers. The first 20 *μ*s of CG-MD (sampled every 750 ps, see Supplementary Fig. 16) contain the equilibration phase, while the CG-MD from 20 to 40 *μ*s is employed for the study in equilibrium conditions (sampling every 300 ps).

For the **BTA**_*w*_ model, we performed a CG-MD simulation at *T* = 300 K, of 40 *μ*s, starting from randomly dispersed monomers. The analysis reported in Supplementary Fig. 32f, g) is performed on the equilibrium trajectory from 20 to 40 *μ*s (sampling every 300 ps). The **BTA** and **BTA**_*w*_ 40-monomer fibre simulations of Supplementary Fig. 32c–e are performed starting from a 40-monomer assembly configuration obtained with the *Fibre* **3** model of Ref. ^49^ and extended for 10 *μ*s of GC-MD.

### Analysis of the CG-MD simulations

All the analyses were carried out by means of python scripts employing the MD-Analysis package^65,66^. Exceptions are the analyses reported in Figs. 2c, 4d, Supplementary Fig. 8, Supplementary Figs. 9 and 10 and the analysis reported in Supplementary Fig. 1, which were performed using, respectively, the *clustsize* and *rdf* GROMACS tools, and the analysis of Supplementary Fig. 32c–e, which was performed using PLUMED^67^ (version 2.7) and the python library Scikit-learn^68^. The snapshots in Figs. 3c, 2a, b and Supplementary Fig. 11 were rendered with VMD ^69^.

For the analyses of monomer aggregation, we proceeded as follows: two **M** monomers were considered in contact (*i.e*., they belonged to the same assembly) if their core beads lied within a distance of *r*_cut_ = 0.6 nm; we chose this value for *r*_cut_ as this radius includes the first peak of the radial distribution function (*g(r)*) (Supplementary Fig. 1). For the **BTA** and **BTA**_*w*_ models, the contact radius *r*_cut_ has been fixed at 0.6 nm, obtained following the same criterion. We also tested how a different choice of the cutoff can impact the analysis (*vide infra*).

The molecular traffic and flux analyses^55^, monitoring the dynamic behaviour of the system, were performed both for equilibration part (the first 20 *μ*s) and for the equilibrium part of the trajectories (from 20 to 40 *μ*s, see Figs. 2d bottom, f and 4e). Both quantities are cumulative, *i.e*., they keep track of the previous behaviour of the system and are defined as:

1 $$T(\tau )=\mathop{\sum }\limits_{t=0}^{\tau }\frac{b(t)+u(t)}{N}\qquad F(\tau )=\mathop{\sum }\limits_{t=0}^{\tau }\frac{b(t)-u(t)}{N},$$

where *N* = 500 is the number of monomers in the system. The traffic (*T*) indicates how many dynamical events (binding *b* and unbinding *u*) per-monomer have occurred until *t* = *τ*, while the flux (*F*) indicates the balance (difference) between such events.

The transition matrices reported in Figs. 3a and 5, record the binding/unbinding events involving each monomer, during the equilibrium phases of CG-MD, i.e., from 20 *μ*s to 40 *μ*s, with a sampling step of Δ*τ* = 300 ps, for all the models. The raw-data transition matrices (central panels in Figs. 3a and 5a, b) report the number of monomer transitions from an assembly of size *i* (row index) to an assembly of size *j* (column index), coloured by a logarithmic scale. Since the system is at the equilibrium, and detailed balance is met, the matrices are symmetric, and the frequency of binding events (upper triangular region of the matrix) equates the frequency of the corresponding unbinding events (lower triangular). We also reported the rate of transitions detected along the 20*μ*s of CG-MD for each of the systems (Supplementary Figs. 21–23).

The transition probability matrices (partially reported in the left and right panels of Figs. 3a and 5a, b), are obtained by normalising the raw-data transition matrices over each row (in such a way that each matrix row sums to 1). In this way, the normalised entries indicate the percentage probabilities that a monomer in a construct of size *i* undergoes transition to a construct of size-range *j* during the sampling step Δ*τ* (these normalised matrices lose the symmetry of the raw transition matrices).

By keeping track of the identity of all individual monomers involved in the sampled transitions (and by reconstructing to which assembly they belong to at each sampling timestep), we could also verify that in the **M** and **BTA** systems monomer/oligomer exchange occurs with higher probability at the fibre tips. In these models, dominated by directional monomer-monomer interactions, exchange events occurring along the backbone of the fibres are negligible (see Supplementary Figs. 21–23 for quantitative details). On the other hand, supramolecular fibres where non-directional solvophobic effects also contribute to the monomer-monomer interactions are populated by bulk defects (e.g., **BTA**_*w*_), which make molecular exchange statistically relevant all along the fibre backbone^49^, as a result of the bulk defects that populate the assembled structures. These results find consistency with the computational^45,58^ and experimental^18^ evidence available for water-soluble BTA fibres.

To classify the different mechanisms of exchange between the self-assembled fibres we divided the entries of the raw transition matrices by their row or column index, depending if they were part of the superior or inferior triangular matrix respectively. In this way we obtained *assembly* transition matrices, which list the number of transitions of an aggregate of size *i* into an aggregate of size *j* (Fig. 3e: left and Supplementary Fig. 25: left column). This holds under the assumption of binary events, *i.e*., the transitions collected in the matrices involve only two assemblies at once. This condition is not strictly satisfied, but we verified that the qualitative message of the outcome is robust in this sense (by computing the transition matrices with a different sampling step Δ*τ* and contact radius *r*_cut_, see below).

We facilitated the interpretation of assembly transition matrices via the illustrative scheme/legend of Fig. 3d: the matrix is divided into four areas, which identify the class of transition events based on two characteristic aggregate sizes, *A* = 〈size〉 and *E* = *A*/5, related to the typical size of the formed aggregates, as explained in the main text. We then computed the probability of each class of events by summing all the events included in each region, and dividing this number by the total number of transition events recorded in the matrix (i.e., the sum of all the entries except the diagonal). This provided the probabilities reported in the main text and the matrices depicted in Fig. 3e, Supplementary Figs. 15, 25 and  32e, g.

To further investigate the dependence of the results changing the spatial and temporal resolution, we computed the statistical weight of different polymerisation/depolymerisation mechanisms in the **M** and **BTA** models (the percentage associated to the areas defined in Fig. 3d) by changing the contact cutoff radius *r*_cut_ and the sampling step Δ*τ*. Firstly, we analysed the transitions detected by using *r*_cut_ = 0.7 nm. We obtained the results reported in Supplementary Fig. 26, where the probabilities show a similar trend to that obtained with the 0.6 nm cutoff radius used in the rest of the analyses (Supplementary Fig. 25), for both the **M** and the **BTA** systems. Then, changing the temporal resolution of the sampling, *i.e*. increasing the sampling step to Δ*τ* = 3 ns, we computed the transition matrices for both the **M** and the **BTA** systems (see Supplementary Figs. 28 and 29) and the probabilities associated to the different areas of the assembly transition matrices, obtaining the results reported in Supplementary Fig. 30. Also these additional analyses show a trend similar to the original ones for both the **M** and the **BTA** systems, demonstrating the robustness of our conclusions.

The structural analysis of the 40-monomer fibres, reported in Supplementary Fig. 32c–e, mirrors the approach used in Ref. ^49^ to study explicit solvent BTA-like models.

## Supplementary information

Supplementary Information

## Data Availability

Details on the procedures for the parametrization of the molecular models and on the simulations’ setup, along with additional simulation data, are provided in the Methods section and in the Supplementary Information file. Complete data and materials pertaining to the molecular simulations conducted herein (input files, model files, raw data, analysis tools, etc.) are available at: 10.5281/zenodo.6453179. Other information needed is available from the corresponding author upon request.
