# Supplementary material for: Molecular communications in complex systems of dynamic supramolecular polymers
Source: Nat Commun. 2022 Apr 20;13:2162. doi: 10.1038/s41467-022-29804-5 (PMC9021206; doi:10.1038/s41467-022-29804-5)
Supplement: Supplementary file 1 — Supplementary Information [file 41467_2022_29804_MOESM1_ESM.pdf]

Supplementary Information: Molecular Communications in Complex Systems of Dynamic Supramolecular Polymers

Martina Crippa[1], Claudio Perego[2], Anna L. de Marco[2,3], and Giovanni M. Pavan[1,2,*]

[1]Department of Applied Science and Technology, Politecnico di Torino, Corso Duca degli Abruzzi 24, 10129 Torino, Italy
[2]Department of Innovative Technologies, University of Applied Sciences and Arts of Southern Switzerland, Polo Universitario Lugano, Campus Est, Via la Santa 1, 6962 Lugano-Viganello, Switzerland
[3]Department of Physics, Università degli Studi di Genova, Via Dodecaneso 33, 16100 Genova, Italy
[*]corresponding author: Giovanni M. Pavan (giovanni.pavan@polito.it)

Supplementary Figures

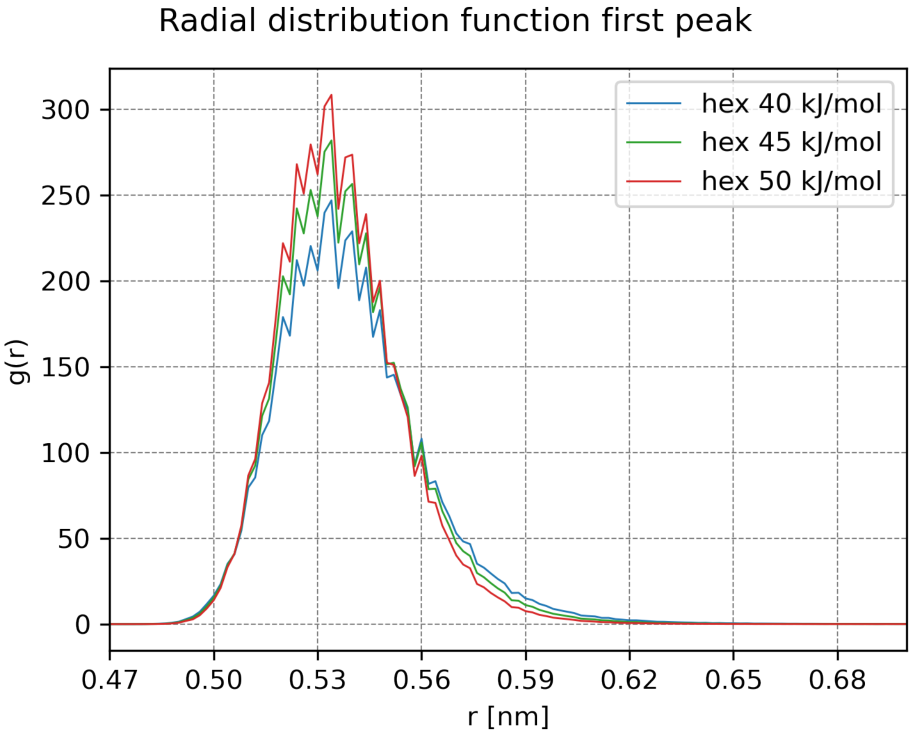

Supplementary Figure 1: First peak of the radial distribution function for the equilibrium part of three different **M** systems ($\epsilon = 40\,\mathrm{kJ\,mol^{-1}},\ 45,\mathrm{kJ\,mol^{-1}}$ and $50\,\mathrm{kJ\,mol^{-1}}$).

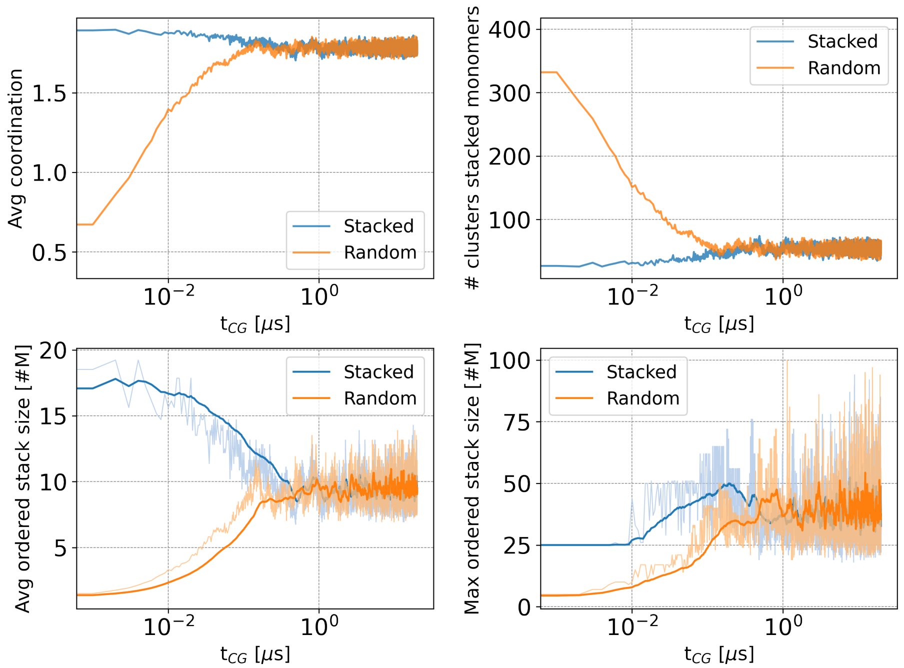

Supplementary Figure 2: Self-assembly observables for the R and S systems interacting with $\epsilon = 40\,\mathrm{kJ\,mol^{-1}}$: average coordination (top left), number of assemblies (top right), average size of assemblies (bottom left), size of the largest assembly (bottom right).

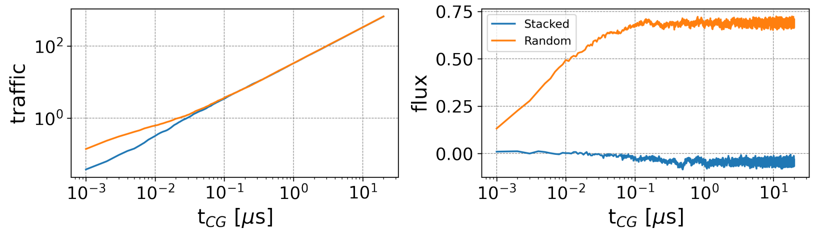

Supplementary Figure 3: Traffic and flux for the R and S systems interacting with $\epsilon = 40\,\mathrm{kJ\,mol^{-1}}$.

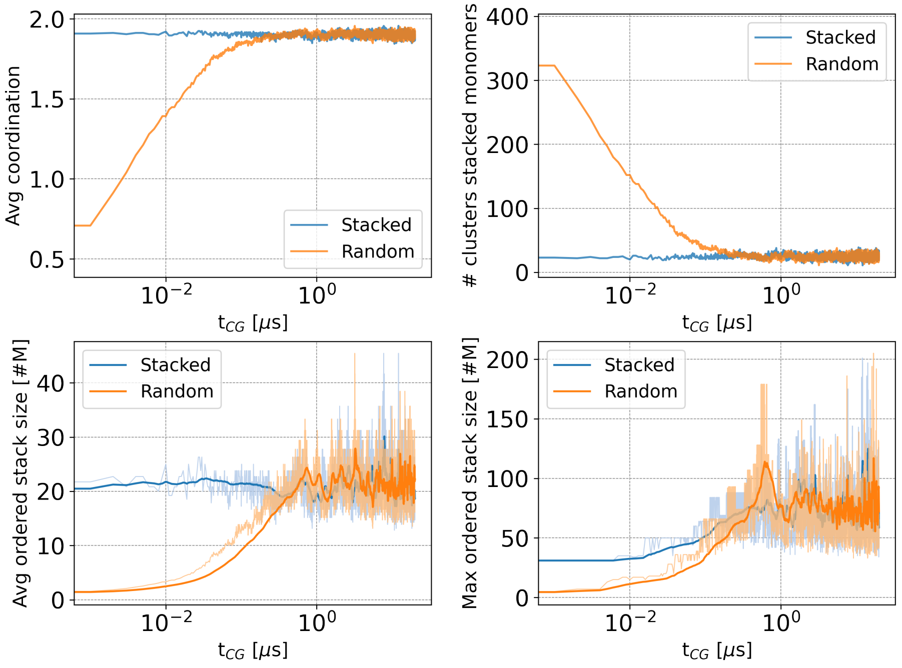

Supplementary Figure 4: Self-assembly observables for the R and S systems interacting with $\epsilon = 45\,\mathrm{kJ\,mol^{-1}}$: average coordination (top left), number of assemblies (top right), average size of assemblies (bottom left), size of the largest assembly (bottom right).

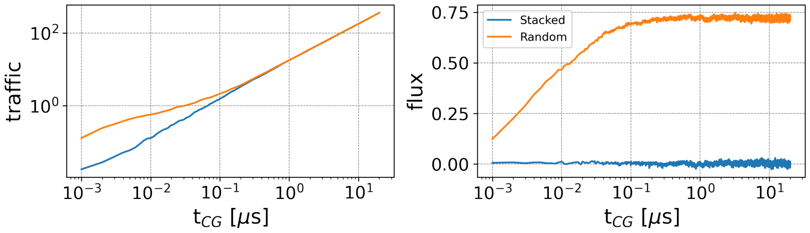

Supplementary Figure 5: Traffic and flux for the R and S systems interacting with $\epsilon = 45\,\mathrm{kJ\,mol^{-1}}$.

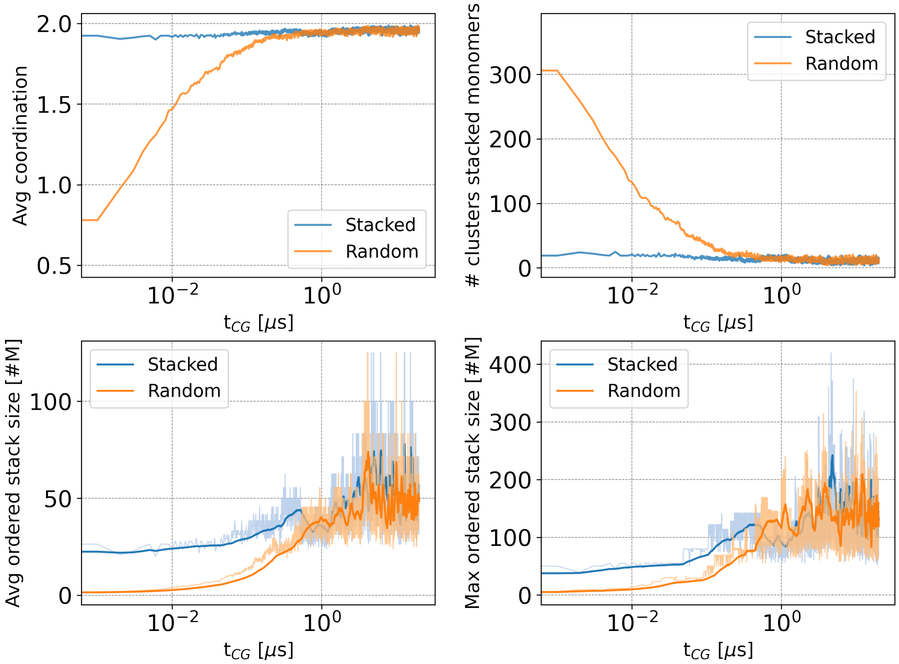

Supplementary Figure 6: Self-assembly observables for the R and S systems interacting with $\epsilon = 50\,\mathrm{kJ\,mol^{-1}}$: average coordination (top left), number of assemblies (top right), average size of assemblies (bottom left), size of the largest assembly (bottom right).

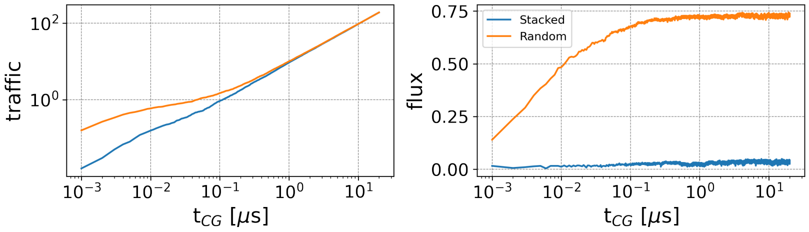

Supplementary Figure 7: Traffic and flux for the R and S systems interacting with $\epsilon = 50\,\mathrm{kJ\,mol^{-1}}$.

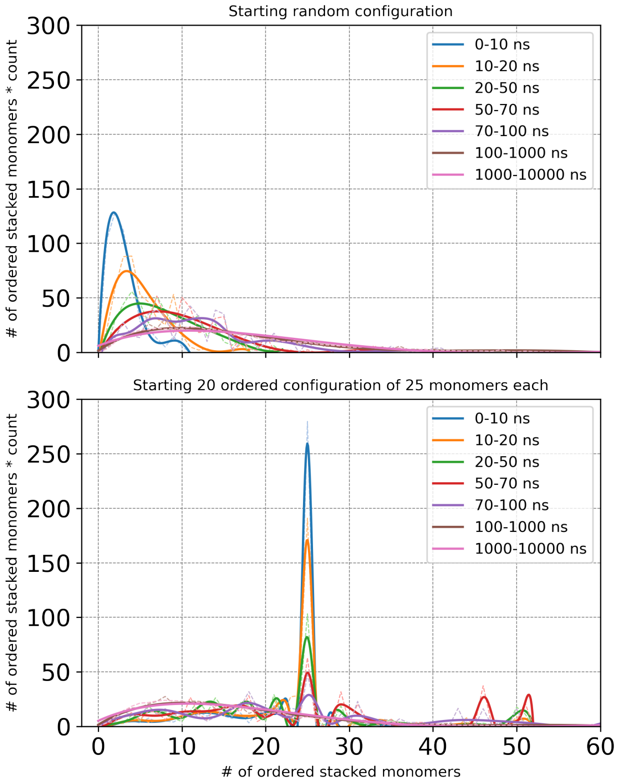

Supplementary Figure 8: Distribution of monomers into aggregates of different sizes computed over different time intervals of the CG-MD trajectory, comparing R (top) and S (bottom) systems interacting by $\epsilon = 40\,\mathrm{kJ\,mol^{-1}}$. Comparing the two panels, convergence between the S and R system population distributions after $t_1 = 1\,\mu\mathrm{s}$ (pink curve) is observed.

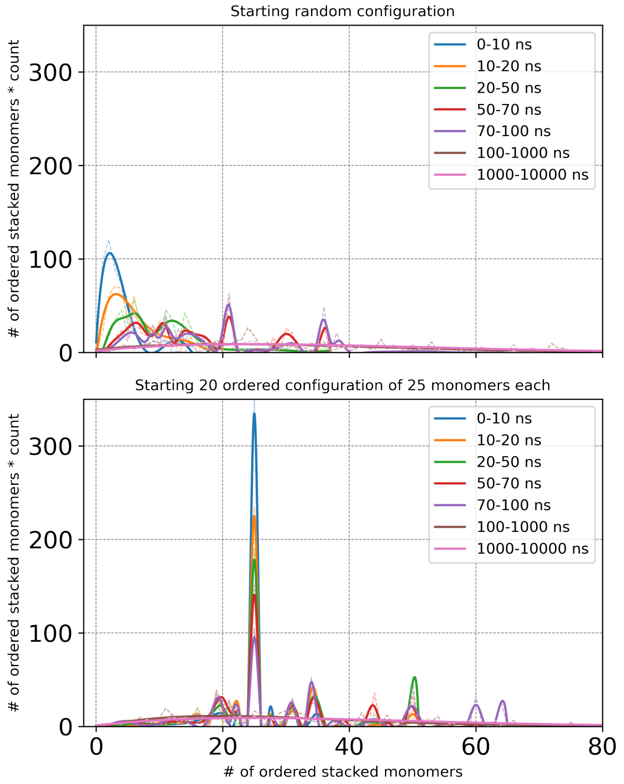

Supplementary Figure 9: Distribution of monomers into aggregates of different sizes computed over different time intervals of the CG-MD trajectory, comparing R (top) and S (bottom) systems interacting by $\epsilon = 45\,\mathrm{kJ\,mol^{-1}}$. Comparing the two panels, convergence between the S and R system population distributions after $t_1 = 1\,\mu\mathrm{s}$ (pink curve) is observed.

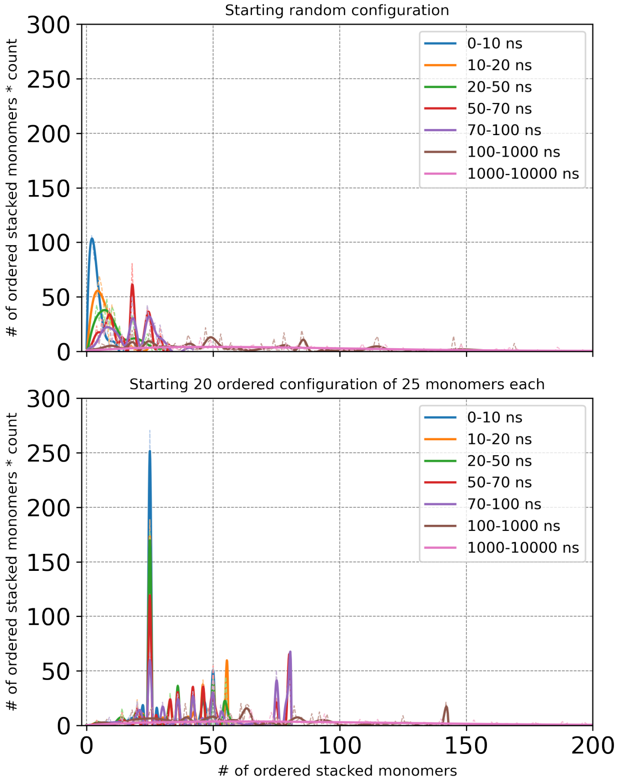

Supplementary Figure 10: Distribution of monomers into aggregates of different sizes computed over different time intervals of the CG-MD trajectory, comparing R (top) and S (bottom) systems interacting by $\epsilon = 50\,\mathrm{kJ\,mol^{-1}}$. Comparing the two panels, convergence between the S and R system population distributions after $t_1 = 1\,\mu s$ (pink curve) is observed.

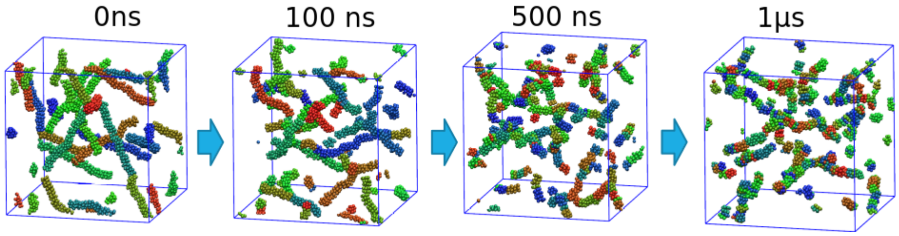

Supplementary Figure 11: Snapshots of the S system ($\epsilon = 40\,\mathrm{kJ\,mol^{-1}}$) at different times: at time t_0 each fibre is colored with a different color. During the MD the fibres exchange monomers and after $t_1 = 1\,\mu s$ the new fibres are a mixture of the initial fibres.

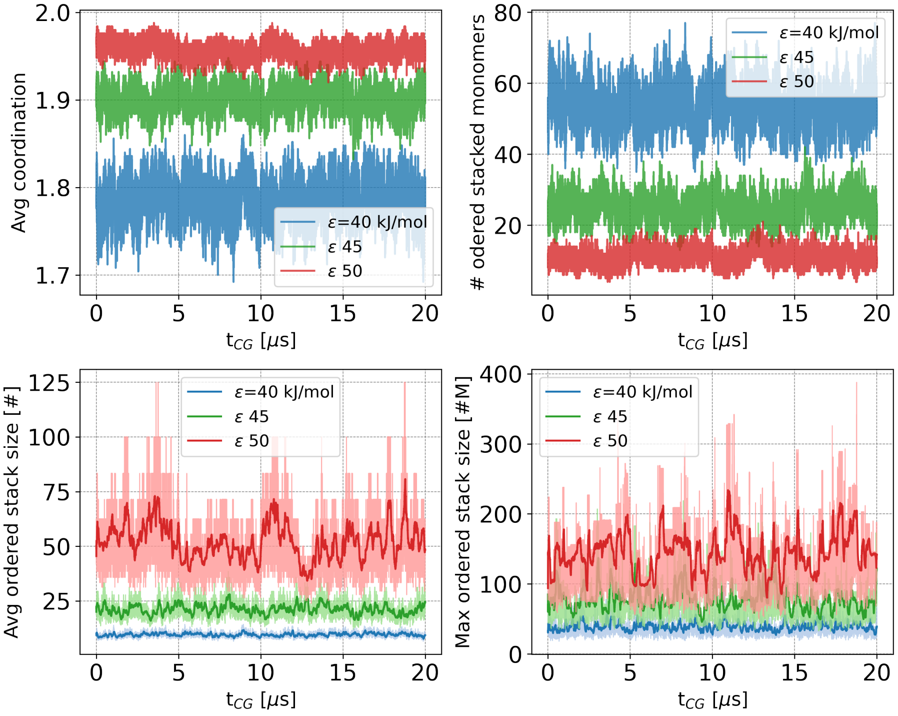

Supplementary Figure 12: Self-assembly observables comparison between $\epsilon = 40\,\mathrm{kJ\,mol^{-1}}$, $45\,\mathrm{kJ\,mol^{-1}}$ and $50\,\mathrm{kJ\,mol^{-1}}$ **M** systems at the equilibrium: average coordination (top left), number of assemblies (top right), average size of assemblies (bottom left), size of the largest assembly (bottom right).

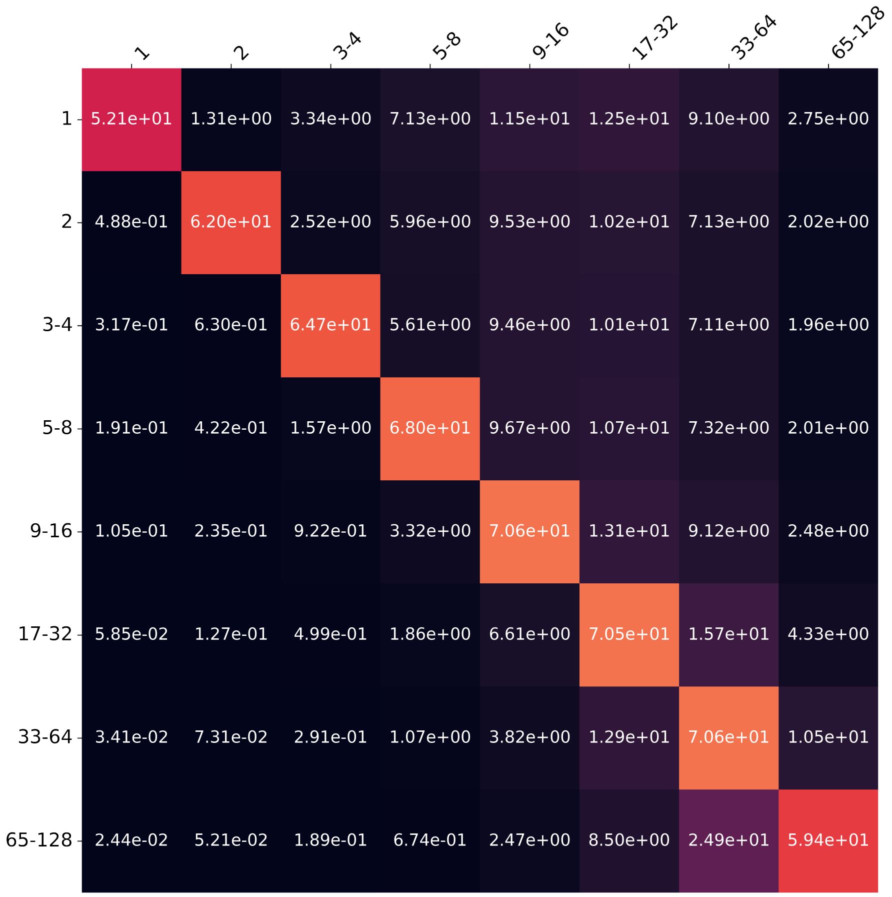

Supplementary Figure 13: Sub-section of the probability transition matrix (same as reported in Figure 3a, left panel). The percentage probabilities are reported using scientific notations with three significant digits: in the main text the probabilities < 0.5 are rounded to zero.

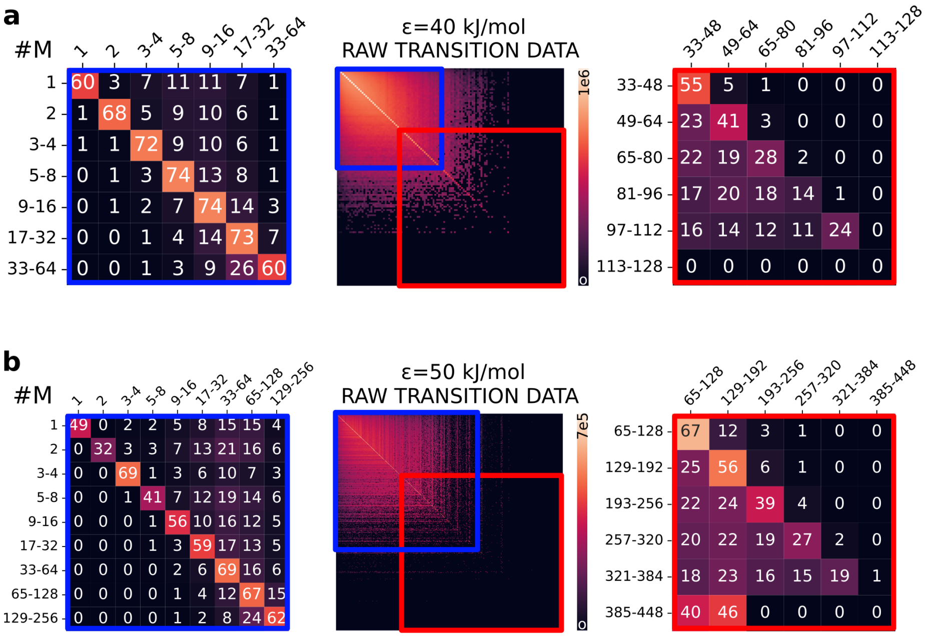

Supplementary Figure 14: Transition matrices for the M-model. (a) $\epsilon = 40\,\mathrm{kJ\,mol^{-1}}$ and (b) $\epsilon = 50\,\mathrm{kJ\,mol^{-1}}$. Each entry (i, j) of the raw transition matrices (central column) shows how many monomers transit from an assembly of size i to an assembly of size j every $\Delta\tau = 300\,\mathrm{ps}$ of CG-MD time; The left and right panels report two sub-regions of the transition probability matrix (red and blue rectangles). Here, the size of the aggregates are grouped for clarity. The numbers in the cells indicate the percentage probability (the 0s identify transitions with probability < 0.5). The raw transition matrices are colored in log scale.

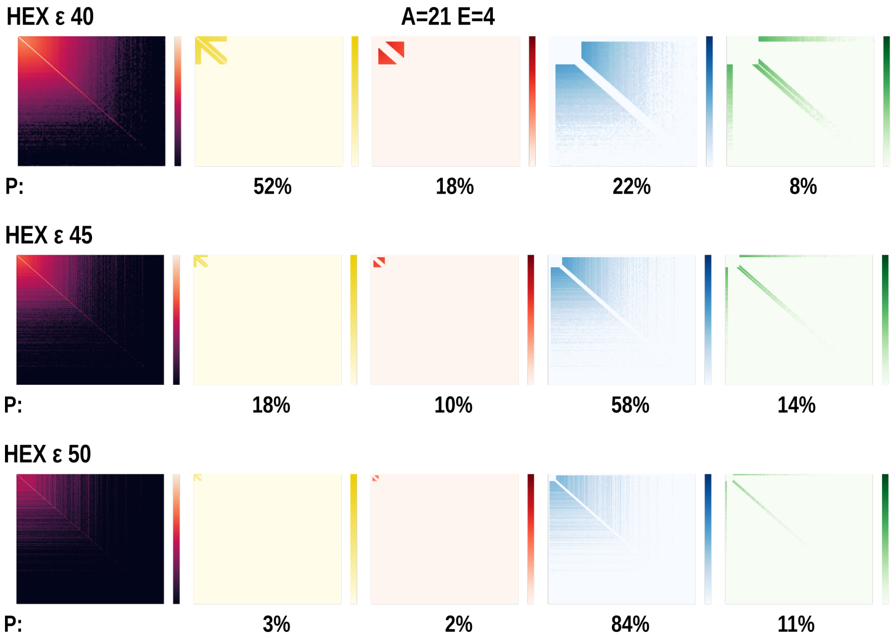

Supplementary Figure 15: Assembly transition matrices for the **M** systems (left), decomposed into areas identifying different classes of polymerisation/depolymerisation mechanisms (see Methods for details). The areas are defined by the parameters $A = 21$ and $E = \langle A \rangle / 5 \approx 4$, as explained in the main text. The percentage is computed as the sum of each entry of the matrix in the considered area divided by the sum of all the entries of the matrix, without considering the diagonal: this gives an estimate of the predominant mechanism by which the system communicates. The obtained percentage are reported under each areas.

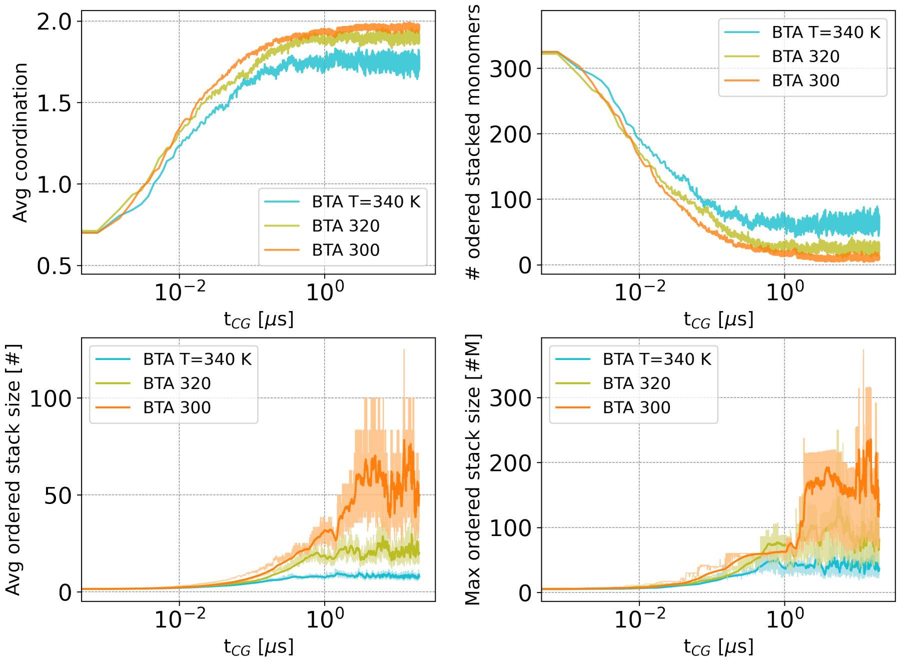

Supplementary Figure 16: Self-assembly observables comparison between $T = 340\,\mathrm{K}$, $T = 320\,\mathrm{K}$ and $T = 300\,\mathrm{K}$ **BTA** system starting from random conformations in log scale: average coordination (top left), number of assemblies (top right), average size of assemblies (bottom left), size of the largest assembly (bottom right).

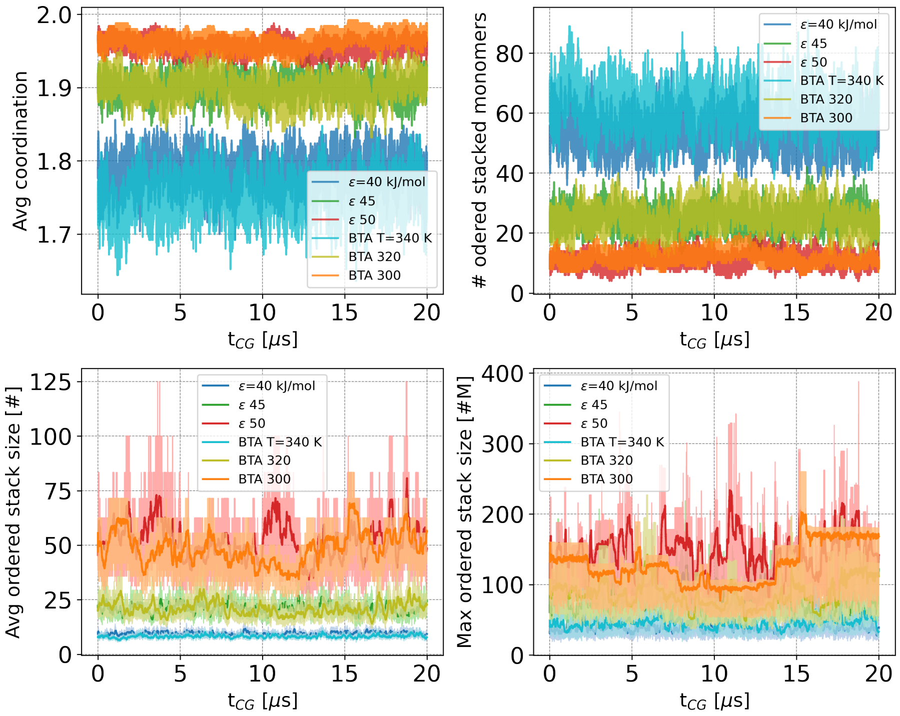

Supplementary Figure 17: Self-assembly observables comparison between $T = 340\,\mathrm{K}$, $T = 320\,\mathrm{K}$ and $T = 300\,\mathrm{K}$ **BTA** system and $\epsilon = 40\,\mathrm{kJ\,mol^{-1}}$, $45\,\mathrm{kJ\,mol^{-1}}$ and $50\,\mathrm{kJ\,mol^{-1}}$ **M** systems: average coordination (top left), number of assemblies (top right), average size of assemblies (bottom left), size of the largest assembly (bottom right).

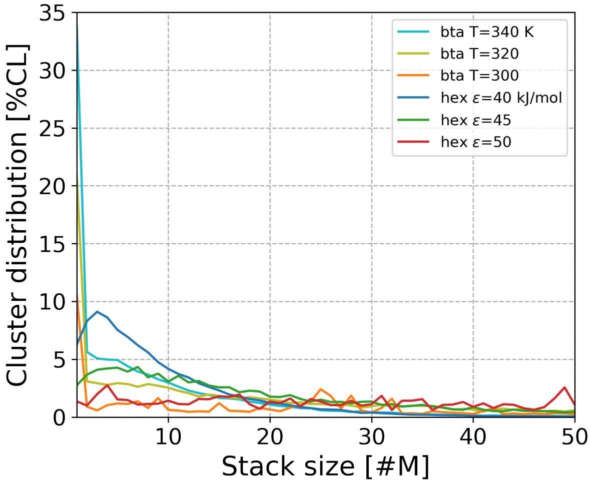

Supplementary Figure 18: Assembly distributions comparison between $\epsilon = 40\,\mathrm{kJ\,mol^{-1}}$, $45\,\mathrm{kJ\,mol^{-1}}$ and $50\,\mathrm{kJ\,mol^{-1}}$ **M** systems and $T = 340\,\mathrm{K}$, $T = 320\,\mathrm{K}$ and $T = 300\,\mathrm{K}$ **BTA** system.

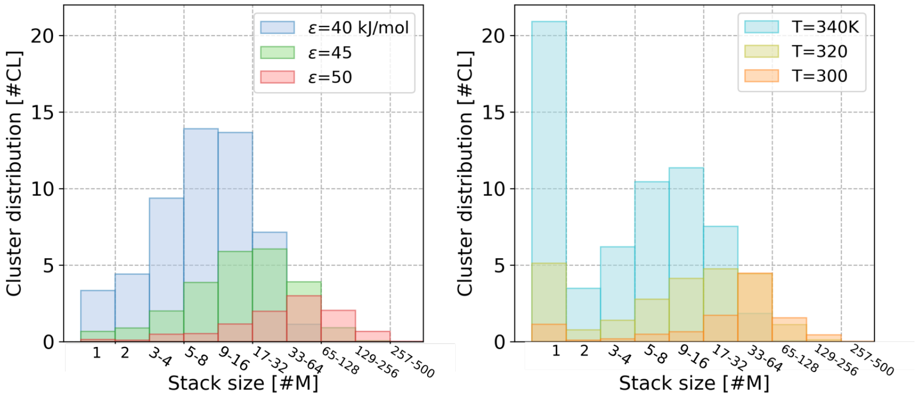

Supplementary Figure 19: Distribution of assemblies of different sizes over the total number of assemblies. Comparison between $\epsilon = 40\,\mathrm{kJ\,mol^{-1}}$, $45\,\mathrm{kJ\,mol^{-1}}$ and $50\,\mathrm{kJ\,mol^{-1}}$ **M** systems (left) and $T = 340\,\mathrm{K}$, $T = 320\,\mathrm{K}$ and $T = 300\,\mathrm{K}$ **BTA** system (right): the sizes are grouped in binary size-ranges and reported in log scale along the x axis.

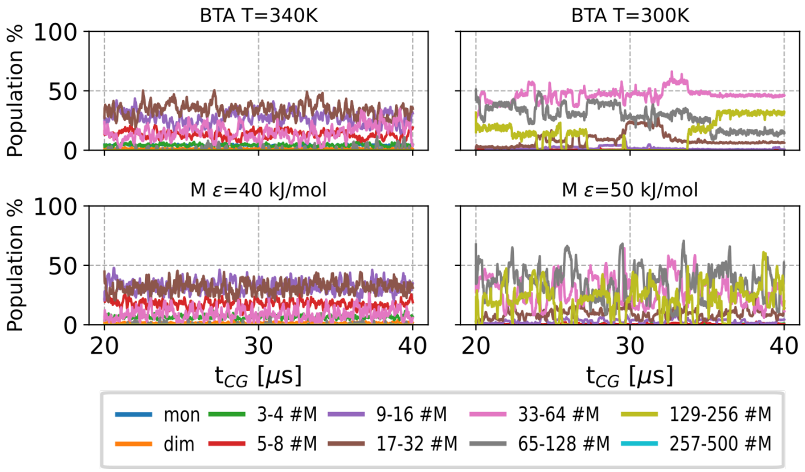

Supplementary Figure 20: Percentage of monomers belonging to different size aggregates for the **BTA** systems at $T = 340\,\mathrm{K}$ (top left) and $T = 300\,\mathrm{K}$ (top right) and **M** system $\epsilon = 40\,\mathrm{kJ\,mol^{-1}}$ (bottom left) and $50\,\mathrm{kJ\,mol^{-1}}$ (bottom right).

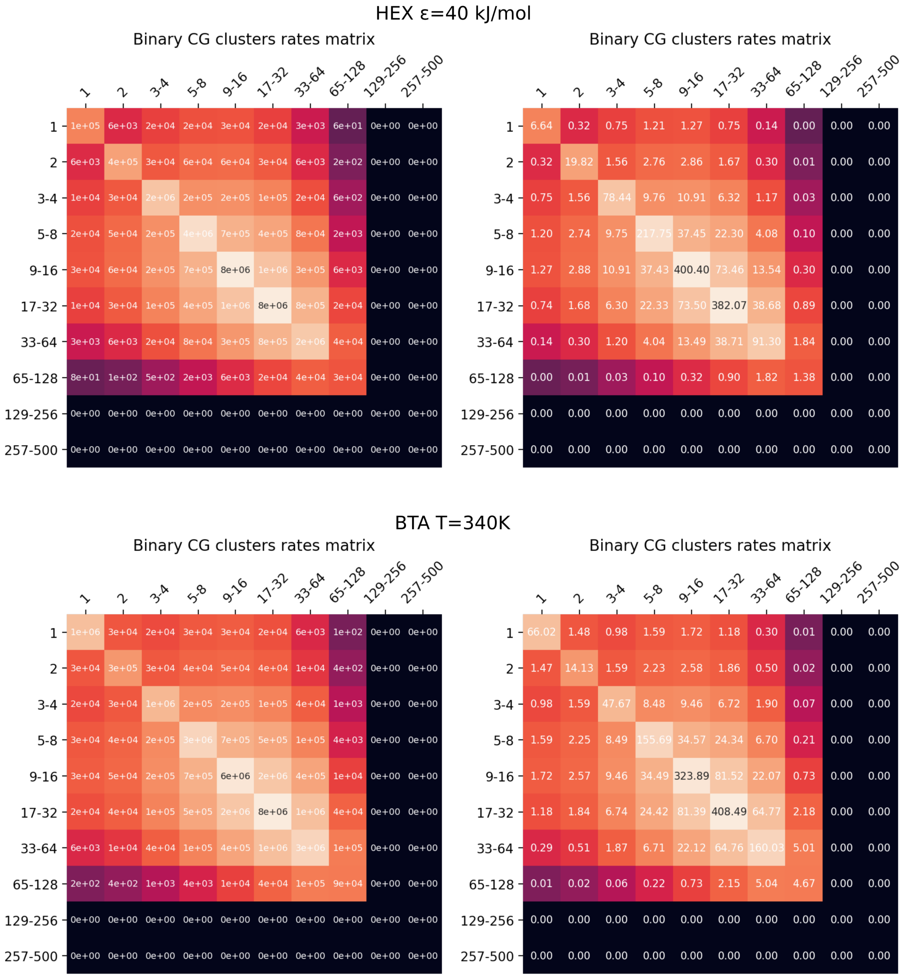

Supplementary Figure 21: **M** with $\epsilon = 40\,\mathrm{kJ\,mol^{-1}}$ (top), and $T = 340\,\mathrm{K}$ (bottom): raw transition matrices grouped in binary size-ranges (left) and transition rate matrices in ns^{-1}, obtained from raw data matrices divided by the trajectory time-length (right), both colored in log scale. During the simulations we sampled a total of 1.4×10^7 and 1.8×10^7 monomer transitions between aggregates of different size (off-diagonal entries) for the **M** and **BTA** models respectively, $3 \times 10^{-4}\%$ and $9 \times 10^{-4}\%$ of which can be considered as exchange of monomers/oligomers from the bulk of existing fibres. This indicates that in both systems $> 99\%$ of transition events involves exchange at the fibres tips.

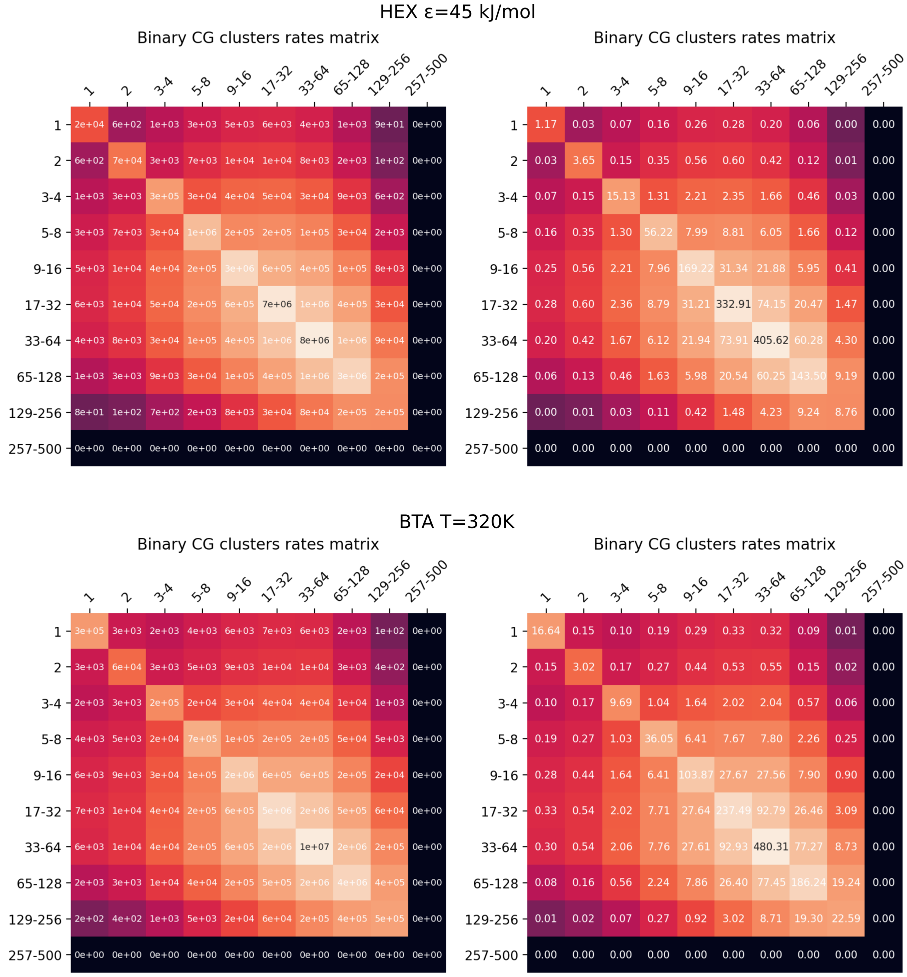

Supplementary Figure 22: **M** with $\epsilon = 45\,\mathrm{kJ\,mol^{-1}}$ (top), and $T = 320\,\mathrm{K}$ (bottom): raw transition matrices grouped in binary size-ranges (left) and transition rate matrices in ns^{-1}, obtained from raw data matrices divided by the trajectory time-length (right), both colored in log scale. During the simulations we sampled a total of 1.7×10^7 and 2.1×10^7 monomer transitions between aggregates of different size (off-diagonal entries) for the **M** and **BTA** models respectively, $1 \times 10^{-4}\%$ and $3 \times 10^{-4}\%$ of which can be considered as exchange of monomers/oligomers from the bulk of existing fibres ($> 99\%$ of transition events involve exchange at the fibres tips, in both systems).

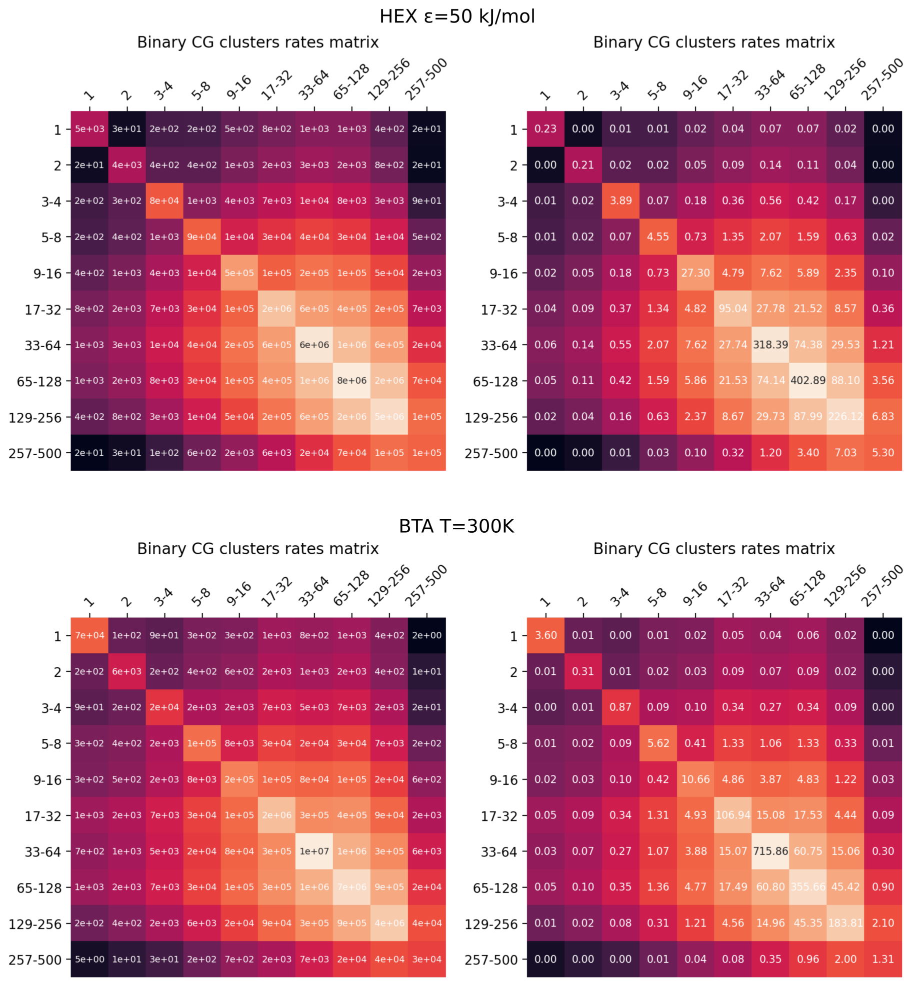

Supplementary Figure 23: **M** with $\epsilon = 50\,\mathrm{kJ\,mol^{-1}}$ (top), and $T = 300\,\mathrm{K}$ (bottom): raw transition matrices grouped in binary size-ranges (left) and transition rate matrices in ns^{-1}, obtained from raw data matrices divided by the trajectory time-length (right), both colored in log scale. During the simulations we sampled a total of 1.9×10^7 and 1.3×10^7 monomer transitions between aggregates of different size (off-diagonal entries) for the **M** and **BTA** models respectively, 4×10^{-5}% and 9×10^{-5}% of which can be considered as exchange of monomers/oligomers from the bulk of existing fibres ($>$ 99% of transition events involve exchange at the fibres tips, in both systems).

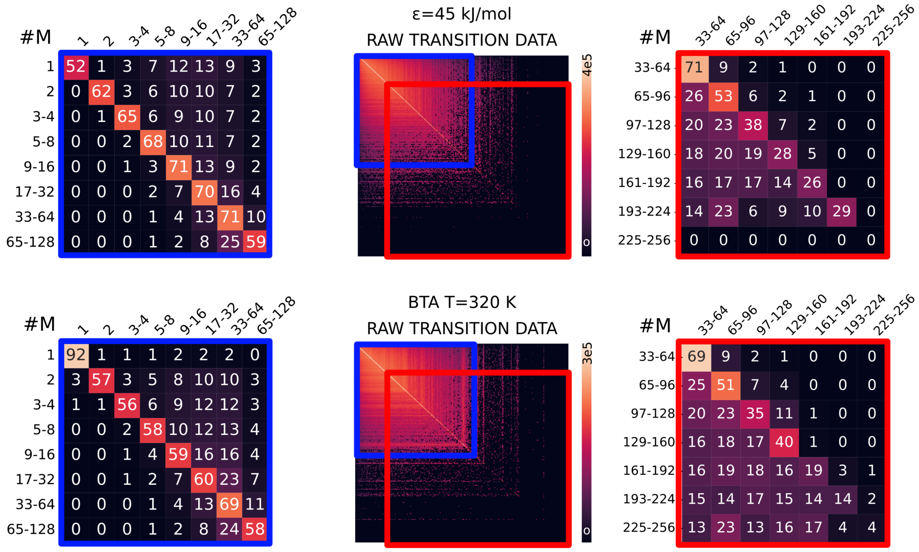

Supplementary Figure 24: Comparison between **BTA** $T = 320\,\mathrm{K}$ (bottom) and **M** with $\epsilon = 45\,\mathrm{kJ\,mol^{-1}}$(top). The raw transition matrix (center) and transition probability sub-matrices (left and right panels, the blue and red rectangles indicate the region highlighted) are reported. Here, the size of the aggregates are grouped for clarity. The numbers in the cells indicate the percentage probability (the 0s identify transitions with probability < 0.5). The transition matrices are colored in log scale.

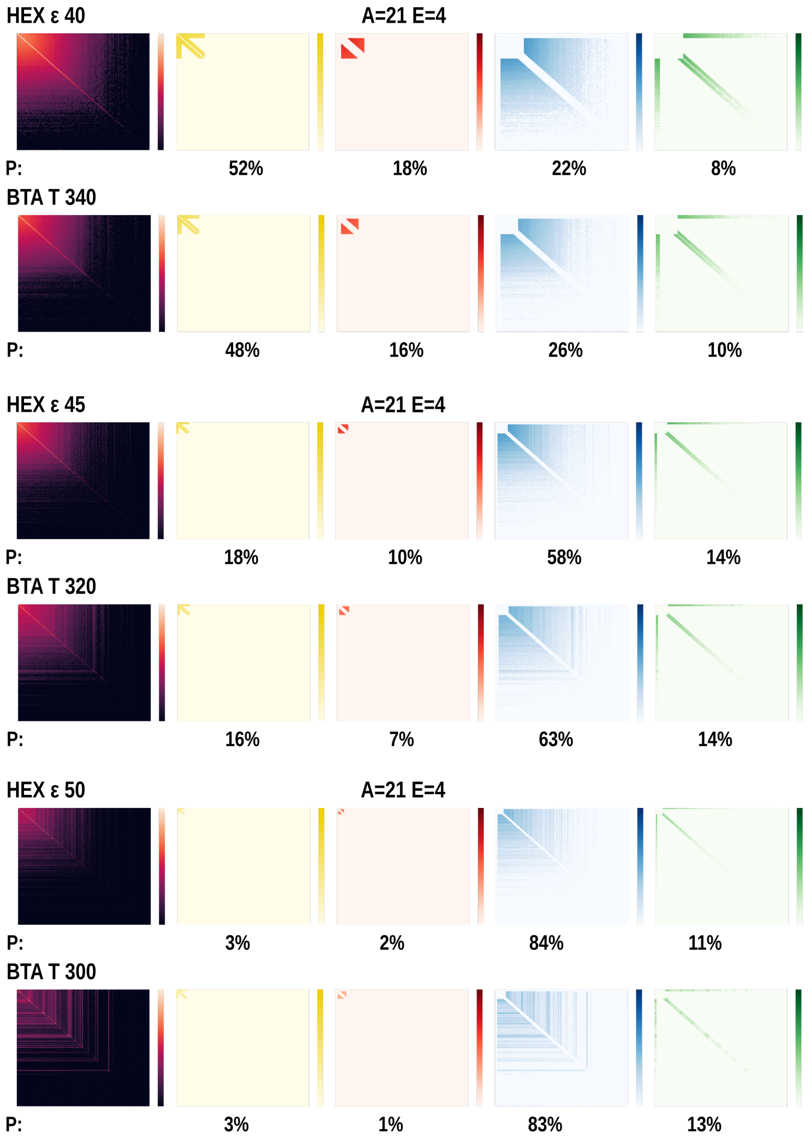

Supplementary Figure 25: Assembly transition matrices (see Methods for details): comparison between **M** system and **BTA** system for $A = 21$ and $E = \langle A \rangle / 5 \approx 4$.

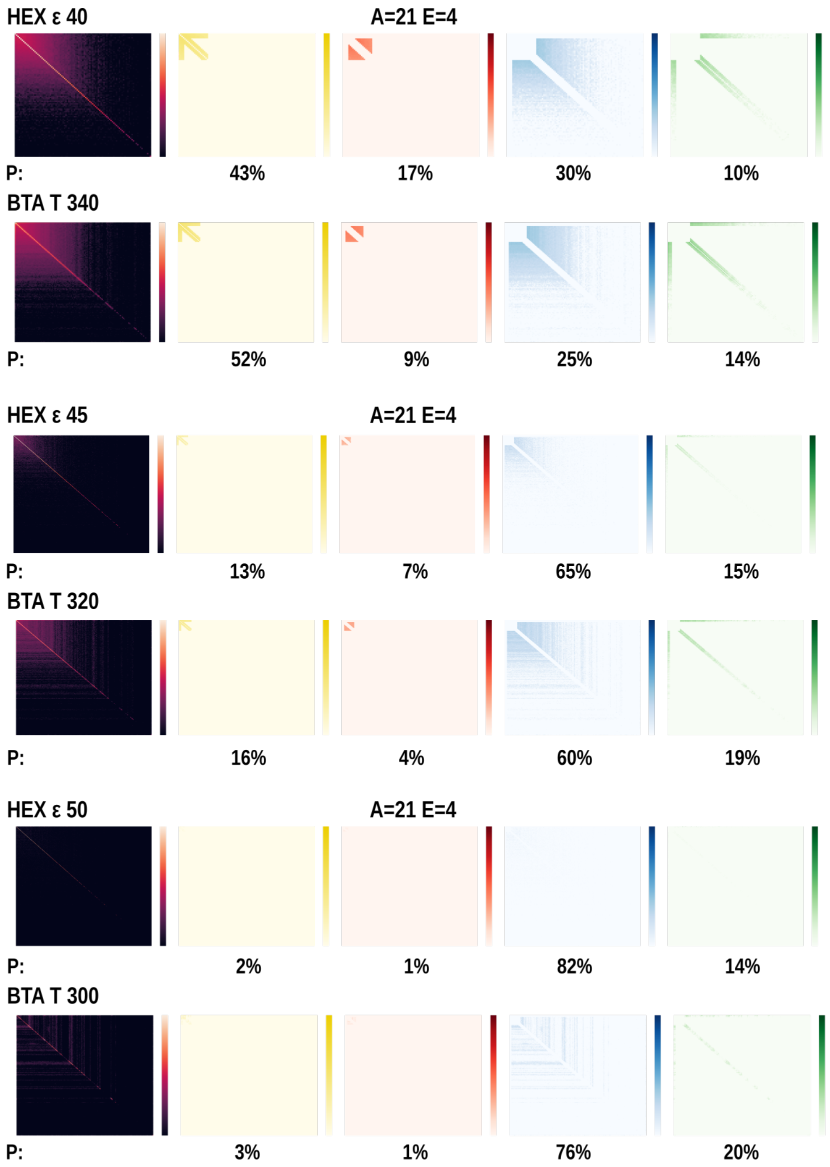

Supplementary Figure 26: Assembly transition matrices (see Methods for details): comparison between **M** system and **BTA** system for $A = 21$ and $E = \langle A \rangle/5 \approx 4$ with cut-off radius $r_{\rm rcut} = 0.7$nm.

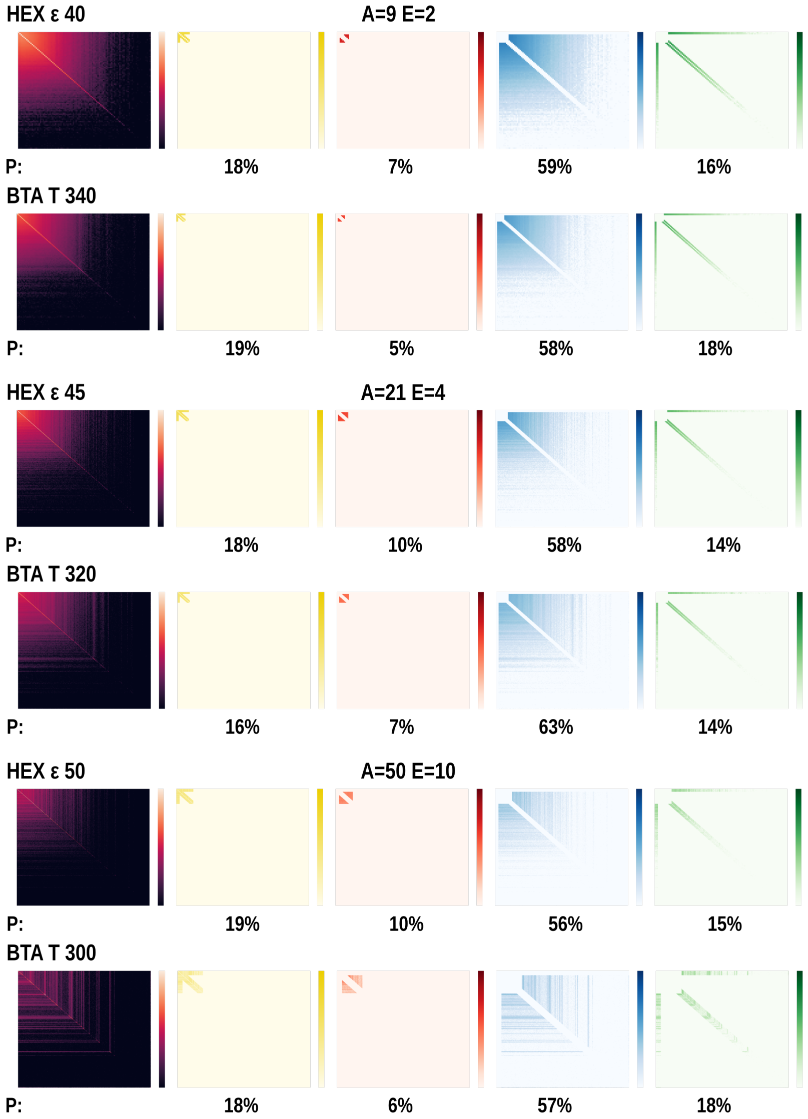

Supplementary Figure 27: Assembly transition matrices (see Methods for details): comparison between **M** system and **BTA** system for $A = \langle \text{size} \rangle$, namely the average size relative to the system under study and $E = \langle A \rangle / 5$.

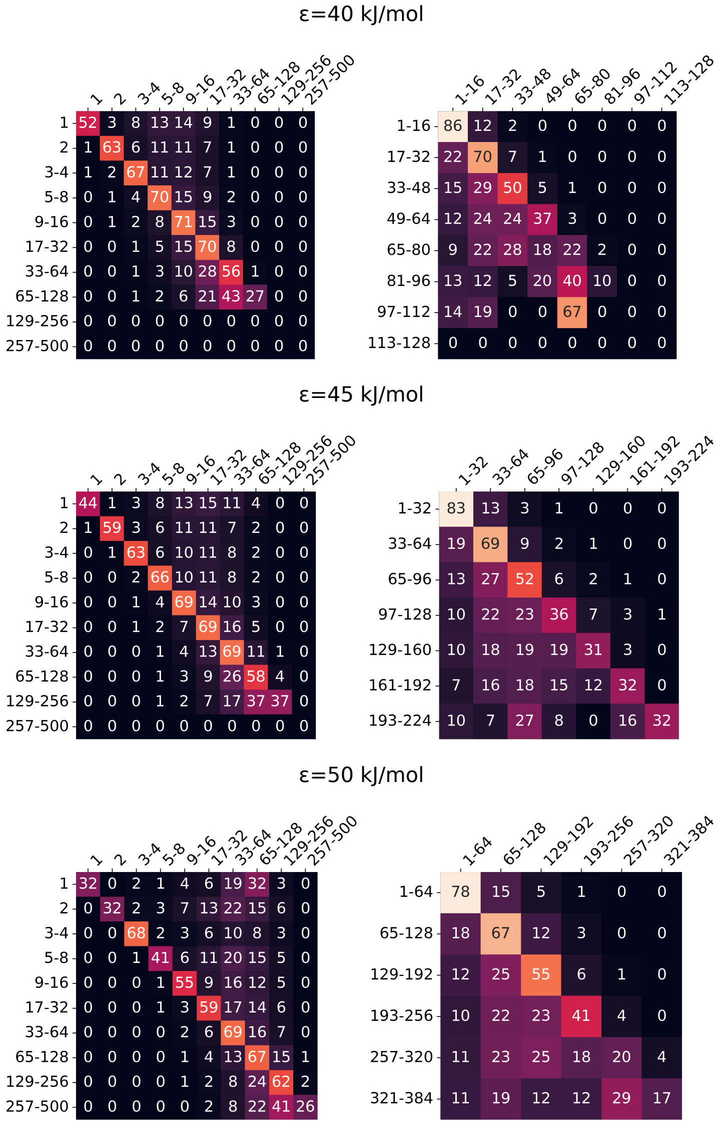

Supplementary Figure 28: Probability transition matrices for **M** systems sampled every $\Delta\tau = 3\,\text{ns}$ and cut-off $r_{\text{cut}} = 0.6\,\text{nm}$. The data is grouped in size-ranges (binary and regular subdivision in the left and right panels, respectively).

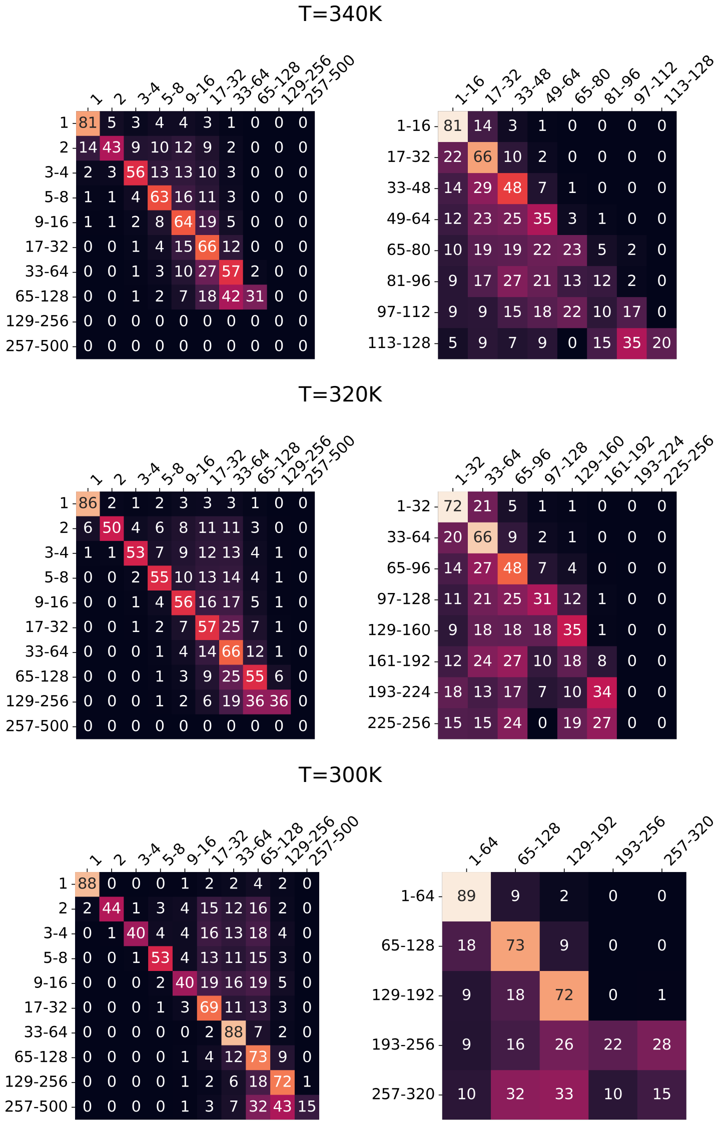

Supplementary Figure 29: Probability transition matrices for **BTA** systems sampled every $\Delta\tau = 3\,\text{ns}$ and cut-off $r_{\text{cut}} = 0.6\,\text{nm}$. The data is grouped in size-ranges (binary and regular subdivision in the left and right panels, respectively).

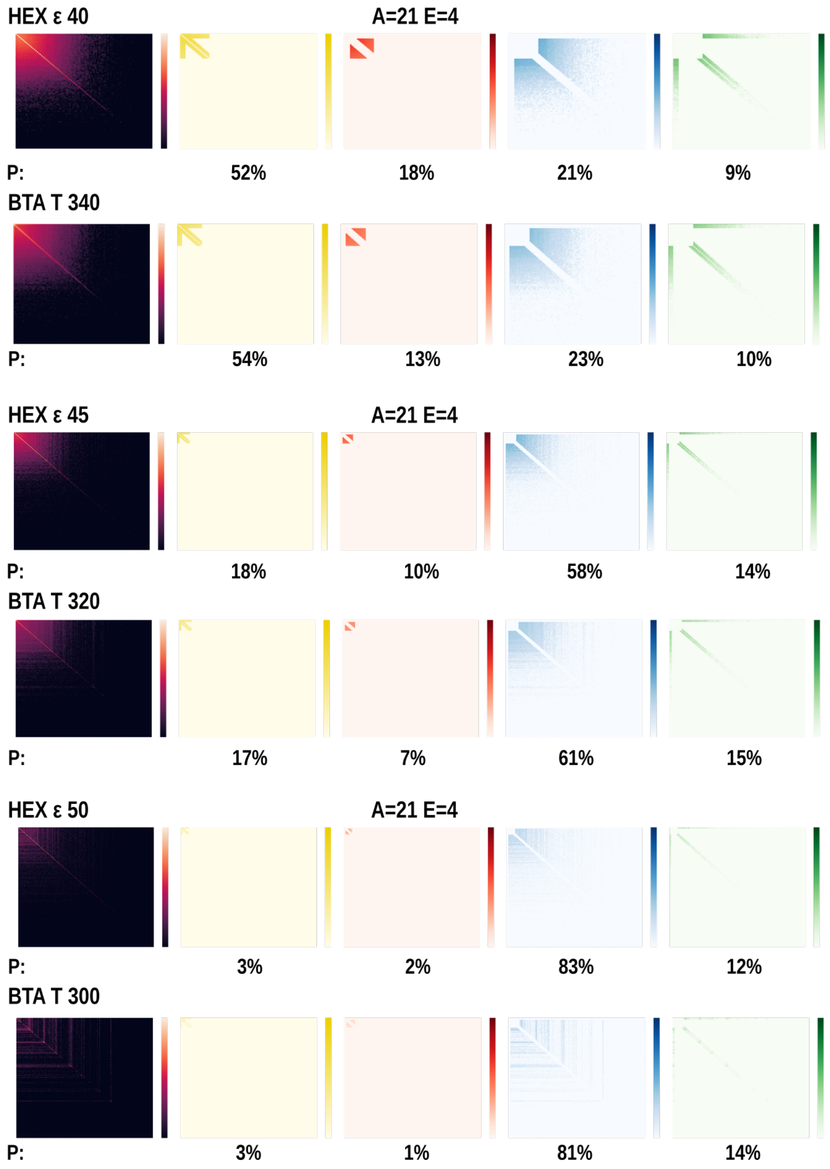

Supplementary Figure 30: Assembly transition matrices (see Methods for details): comparison between **M** system and **BTA** system for $A = 21$ and $E = \langle A \rangle / 5 \approx 4$ sampled every $\Delta\tau = 3\,\text{ns}$ and cut-off radius $r_{\text{cut}} = 0.6\,\text{nm}$.

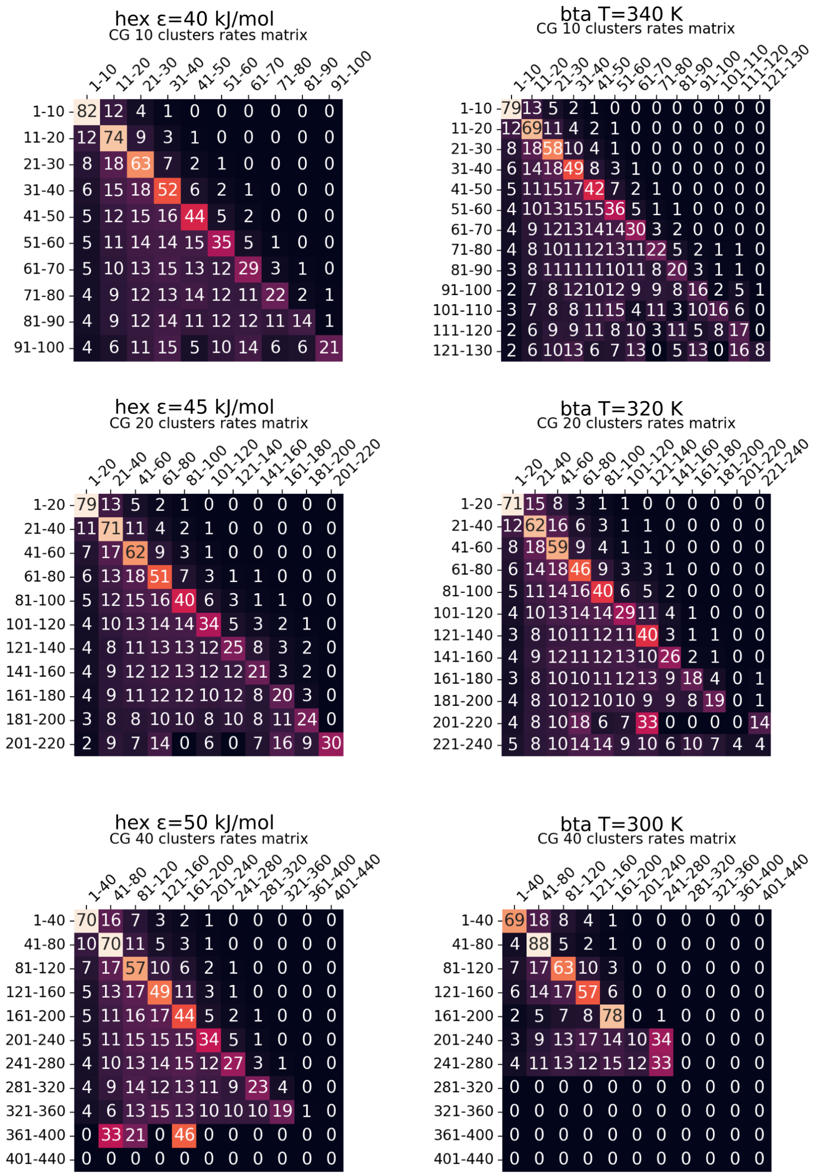

Supplementary Figure 31: Probability transition matrices for **M** and **BTA** systems sampled every $\Delta\tau = 0.3\,\text{ns}$ and cut-off $r_{\text{cut}} = 0.6\,\text{nm}$. The data is grouped in size-ranges (linear size-ranges subdivision).

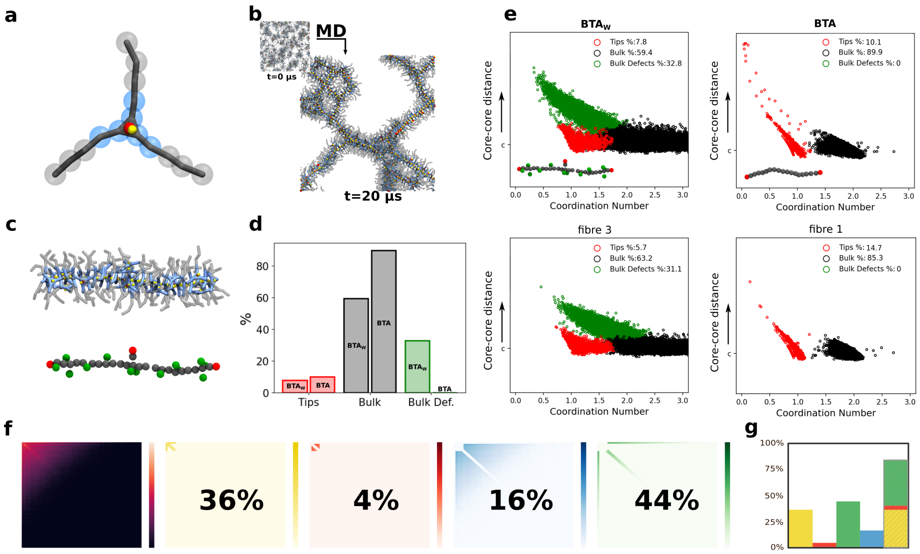

Supplementary Figure 32: **BTA**$_w$ model equilibrium structure and dynamics. (a) CG model of the **BTA**$_w$ monomer: the core monomers (blue beads) interactions are increased to $\epsilon = 4\,\text{kJ}\,\text{mol}^{-1}$, to reproduce the solvophobic effect. (b) Snapshot of **BTA**$_w$ fibres formed spontaneously after $t_{\text{CG}} = 20\,\mu\text{s}$ of CG-MD, starting from 500 dispersed **BTA**$_w$ monomers. (c) A single fibre of 40 **BTA**$_w$ monomers (top) and its core beads colored by their structural configurations (gray: bulk, green: bulk defects and red: tips) (bottom). (d) Comparison of the structural configurations between two fibres of 40 monomers each (**BTA**$_w$ vs **BTA**) obtained by identifying the monomers properties for a $10\mu s$ MD. (e) Comparison among the structure of an equilibrated 40 monomer fibre in: (top-left) the **BTA**$_w$ model; (bottom-left) the *Fibre* **3** explicit solvent model (reported in Ref. 49); (top-right) **BTA** model; (bottom-right) the *Fibre* **1** explicit solvent model (Ref. 49). The scatter plots show the coordination number and the core-core distance (in stacking length units c). The states are divided in clusters (indicated by different colors, as in (c)) according to the monomeric configuration. (f) Assembly transition matrices for the equilibrated 500 monomer **BTA**$_w$ system (left), decomposed into areas identifying different classes of polymerisation/depolymerisation mechanisms (see Methods for details). The areas are defined by the parameters $A = 21$ and $E = \langle A \rangle / 5 \approx 4$, as explained in the main text. The percentage is computed as the sum of each entry of the matrix in the considered area divided by the sum of all the entries of the matrix, without considering the diagonal. The obtained percentage are reported under each areas. (g) Summary of the mechanism probabilities: the mechanism involving small and medium species (yellow, red and green area) are grouped in the last column.

Supplementary Methods

We here report the details on the models and force field parameters used for the **M**, **BTA** and **BTA**$_w$ monomer models, using the GROMACS .itp format for the force field and the GROMACS .gro format for example configurations.

M monomer force-field

```
[ moleculetype ]
; molname       nrexcl
MON               1

[ atoms ]
;id type resnr residu atom cgnr   charge

1   M   1      MONM    M1   1    0
2   P   1      MON     P1   2    0
3   P   1      MON     P2   3    0
4   P   1      MON     P3   4    0
5   P   1      MON     P4   5    0
6   P   1      MON     P5   6    0
7   P   1      MON     P6   7    0

[bonds]
;   i     j   funct   length  force.c.
1     2    1    0.47    20000
1     3    1    0.47    20000
1     4    1    0.47    20000
1     5    1    0.47    20000
1     6    1    0.47    20000
1     7    1    0.47    20000
2     3    1    0.47    20000
3     4    1    0.47    20000
4     5    1    0.47    20000
5     6    1    0.47    20000
6     7    1    0.47    20000
7     2    1    0.47    20000
2     5    1    0.94    15000
3     6    1    0.94    15000
4     7    1    0.94    15000


[constraints]

[angles]
```

```
[dihedrals]

[exclusions]

[ defaults ]
1 2

[ atomtypes ]
; STANDARD types, 4:1 mapping
M    0     72.000      0.000     A   0.0           0.0
P    0     72.000      0.000     A   0.0           0.0


[ nonbond_params ]
; i j   funda c6 c12
; self terms
  M       M  1  4.700000e-01 45.000000e+00  ; dm_rrVIII
  P       P  1  4.700000e-01 0.200000e+00  ; dm_rrVIII
; cross terms
  P       M  1  4.700000e-01 0.200000e+00  ; dm_rrVIII
```

M monomer configuration

```
M Monomer
7
    1MONM    M1    1   0.000   0.000   0.000
    1MON     P1    2   0.470   0.000   0.000
    1MON     P2    3   0.235   0.407   0.000
    1MON     P3    4  -0.235   0.407   0.000
    1MON     P4    5  -0.470   0.000   0.000
    1MON     P5    6  -0.235  -0.407   0.000
    1MON     P6    7   0.235  -0.407   0.000
   5.98336   5.98336   5.98336
```

BTA monomer force-field

```
[moleculetype]
; molname       nrexcl
  BTA  1

[atoms]
; id    type  resnr   residu   atom    cgnr    charge
  1     C1     1       BENZ     M1      1       0
  2     C1     1       BENZ     M2      2       0
  3     C1     1       BENZ     M3      3       0
  4     C1     2        MID     M4      4       0
  5     C1     2        MID     M5      5       0
  6     C1     2        MID     M6      6       0
  7     C1     2       ALKA     C7      7       0
  8     C1     2       ALKA     C8      8       0
  9     C1     2       ALKA     C9      9       0
 10     C1     3       ALKA    C10     10       0
 11     C1     3       ALKA    C11     11       0
 12     C1     3       ALKA    C12     12       0
 13     C1     3       ALKA    C13     13       0
 14     C1     3       ALKA    C14     14       0
 15     C1     3       ALKA    C15     15       0
 16     P5     4        DIP    D16     16       0
 17      D     5         HP    H17     17       1.40
 18      D     5         HN    H18     18      -1.40


[constraints]
  16 17 1       0.14    22000
  16 18 1       0.14    22000

[bonds]
; i  j  funct   length
  1  2  1       0.46    22000
  2  3  1       0.46   22000
  3  1  1       0.46    22000
  1  16 1       0.2656  22000
  2  16 1       0.2656  22000
  3  16 1       0.2656  22000
; BENZ-MID
  1  4  1      0.46     5500
  2  5  1      0.46     5500
  3  6  1       0.46     5500
; MID-MID
  4  7  1       0.46     5500
```

```
  5  8  1       0.46     5500
  6  9  1       0.46     5500
; MID-ALKA
  7 10  1       0.46     5500
  8 11  1       0.46     5500
  9 12  1       0.46     5500
; ALKA-ALKA
 10 13  1       0.46     5500
 11 14  1       0.46     5500
 12 15  1       0.46     5500


[angles]
; i  j  k  funct  angle  force.c.
  3  2  5         2     150     1500
  2  1  4         2     150     1500
  1  3  6         2     150     1500
;
  1  2  5         2     150     1500
  3  1  4         2     150     1500
  2  3  6         2     150     1500
;
  1  4  7       2     180.0 10
  2  5  8       2     180.0 10
  3  6  9       2     180.0 10
;
  4  7 10         2     180.0   10
  5  8 11         2     180.0   10
  6  9 12         2     180.0   10
;
  7 10 13         2     180.0   10
  8 11 14         2     180.0   10
  9 12 15         2     180.0   10
;
 17 16 18         2     180.0   1500

[exclusions]
17 18

[ defaults ]
1 1

[ atomtypes ]
; STANDARD types, 4:1 mapping
P5   72.0 0.000 A 0.0 0.0
C1   72.0 0.000 A 0.0 0.0
```

```
D    24.0 0.000 A 0.0 0.0

[ nonbond_params ]
; i j   funda c6 c12
; self terms
  P5    P5      1   1.07792e-01     1.16191e-03 ; eps=2.5
  C1    C1      1   4.31169e-02     4.64766e-04 ; eps=1.0
  D     D       1   0.00000E-00     0.00000E-00 ; no LJ interaction
; cross terms
  P5    C1      1   2.15584e-02     2.32383e-04 ; eps=0.5
  D     P5      1   0.00000E-00     0.00000E-00 ; no LJ interaction
  D     C1      1   0.00000E-00     0.00000E-00 ; no LJ interaction
```

BTA$_w$ monomer force-field

```
[moleculetype]
; molname       nrexcl
  BTA  1

[atoms]
; id    type  resnr   residu   atom    cgnr    charge
  1     M1     1       BENZ     M1      1       0
  2     M1     1       BENZ     M2      2       0
  3     M1     1       BENZ     M3      3       0
  4     M1     2        MID     M4      4       0
  5     M1     2        MID     M5      5       0
  6     M1     2        MID     M6      6       0
  7     C1     2       ALKA     C7      7       0
  8     C1     2       ALKA     C8      8       0
  9     C1     2       ALKA     C9      9       0
 10     C1     3       ALKA    C10     10       0
 11     C1     3       ALKA    C11     11       0
 12     C1     3       ALKA    C12     12       0
 13     C1     3       ALKA    C13     13       0
 14     C1     3       ALKA    C14     14       0
 15     C1     3       ALKA    C15     15       0
 16     P5     4        DIP    D16     16       0
 17      D     5         HP    H17     17       1.40
```

```
 18      D     5         HN    H18     18       -1.40


[constraints]
  16 17 1       0.14    22000
  16 18 1       0.14    22000

[bonds]
; i  j  funct   length
  1  2  1       0.46    22000
  2  3  1       0.46   22000
  3  1  1       0.46    22000
  1  16 1       0.2656  22000
  2  16 1       0.2656  22000
  3  16 1       0.2656  22000
; BENZ-MID
  1  4  1       0.46     5500
  2  5  1       0.46     5500
  3  6  1       0.46     5500
; MID-MID
  4  7  1       0.46     5500
  5  8  1       0.46     5500
  6  9  1       0.46     5500
; MID-ALKA
  7 10  1       0.46     5500
  8 11  1       0.46     5500
  9 12  1       0.46     5500
; ALKA-ALKA
 10 13  1       0.46     5500
 11 14  1       0.46     5500
 12 15  1       0.46     5500


[angles]
; i  j  k  funct  angle  force.c.
  3  2  5         2     150     1500
  2  1  4         2     150     1500
  1  3  6         2     150     1500
;
  1  2  5         2     150     1500
  3  1  4         2     150     1500
  2  3  6         2     150     1500
;
  1  4  7         2     180.0 10
  2  5  8         2     180.0 10
  3  6  9         2     180.0 10
```

```
;
  4  7 10         2     180.0   10
  5  8 11         2     180.0   10
  6  9 12         2     180.0   10
;
  7 10 13         2     180.0   10
  8 11 14         2     180.0   10
  9 12 15         2     180.0   10
;
 17 16 18         2     180.0   1500

[exclusions]
17 18

[ defaults ]
1 1

[ atomtypes ]
; STANDARD types, 4:1 mapping
P5   72.0 0.000 A 0.0 0.0
C1   72.0 0.000 A 0.0 0.0
M1   72.0 0.000 A 0.0 0.0
D    24.0 0.000 A 0.0 0.0

[ nonbond_params ]
; i j   funda c6 c12
; self terms
  P5    P5      1   1.07792e-01     1.16191e-03 ; eps=2.5
  C1    C1      1   4.31169e-02     4.64766e-04 ; eps=1.0
  M1    M1      1   1.72467e-01     1.85906e-03 ; eps=4
  D     D       1   0.00000E-00     0.00000E-00 ; no LJ interaction
; cross terms
  P5    C1      1   2.15584e-02     2.32383e-04 ; eps=0.5
  P5    M1      1   2.15584e-02     2.32383e-04 ; eps=0.5
  C1    M1      1   2.15584e-02     2.32383e-04 ; eps=0.5
  D     P5      1   0.00000E-00     0.00000E-00 ; no LJ interaction
  D     C1      1   0.00000E-00     0.00000E-00 ; no LJ interaction
  D     M1      1   0.00000E-00     0.00000E-00 ; no LJ interaction

[ defaults ]
1 1

[ atomtypes ]
; STANDARD types, 4:1 mapping
P5   72.0 0.000 A 0.0 0.0
C1   72.0 0.000 A 0.0 0.0
```

```
M1   72.0 0.000 A 0.0 0.0
D    24.0 0.000 A 0.0 0.0

[ nonbond_params ]
; i j   funda c6 c12
; self terms
  P5    P5      1   1.07792e-01     1.16191e-03 ; eps=2.5
  C1    C1      1   4.31169e-02     4.64766e-04 ; eps=1.0
  M1    M1      1   1.72467e-01     1.85906e-03 ; eps=4
  D     D       1   0.00000E-00     0.00000E-00 ; no LJ interaction
  P5    C1      1   2.15584e-02     2.32383e-04 ; eps=0.5
  P5    M1      1   2.15584e-02     2.32383e-04 ; eps=0.5
  C1    M1      1   2.15584e-02     2.32383e-04 ; eps=0.5
  D     P5      1   0.00000E-00     0.00000E-00 ; no LJ interaction
  D     C1      1   0.00000E-00     0.00000E-00 ; no LJ interaction
  D     M1      1   0.00000E-00     0.00000E-00 ; no LJ interaction
```

BTA (and BTA$_w$) monomer configuration

```
BTA
18
    1BENZ   M1     1   1.314  19.651  10.175
    1BENZ   M2     2   1.030  19.440  10.472
    1BENZ   M3     3   1.215  19.879  10.543
    2MID    M4     4   1.545  19.601   9.886
    2MID    M5     5   0.690  19.240  10.500
    2MID    M6     6   1.397  20.158  10.819
    3ALKA   C7     7   1.889  19.852   9.883
    3ALKA   C8     8   0.976  19.040  10.306
    3ALKA   C9     9   1.345  19.875  11.031
    4ALKA   C10   10   1.730  20.057  10.156
    4ALKA   C11   11   1.138  19.169  10.042
    4ALKA   C12   12   1.078  19.631  10.893
    4ALKA   C13   13   1.565  20.281  10.415
    4ALKA   C14   14   1.477  19.228   9.880
    4ALKA   C15   15   0.769  19.336  10.813
    5DIP    D16   16   1.173  19.674  10.385
```

```
  6HP     H17   17   1.317  19.646  10.466
  7HN     H18   18   1.045  19.673  10.329
50.00000  50.00000  50.00000
```
